# Circadian oscillations in chromatin accessibility systematically orchestrate rhythmic gene transcription in soybean

**DOI:** 10.1093/plcell/koag063

**Published:** 2026-03-09

**Authors:** Hao Tian, Zijian Yang, Qichao Lian, Hequan Sun, Xiaomei Zhang, Shuo Liu, Xiaopeng Li, Meihan Ban, Enjie Yu, Changyuan Li, Chunhui Song, Yangbo Chen, Legong Li, Yong-Fu Fu, Liangyu Liu

**Affiliations:** Beijing Key Laboratory of Plant Gene Resources and Biotechnology for Carbon Reduction and Environmental Improvement, and College of Life Sciences, Capital Normal University, Beijing 100048, China; School of Life Sciences and Medicine, Shandong University of Technology, Zibo, Shandong 255000, China; Beijing Key Laboratory of Plant Gene Resources and Biotechnology for Carbon Reduction and Environmental Improvement, and College of Life Sciences, Capital Normal University, Beijing 100048, China; Guangdong Laboratory for Lingnan Modern Agriculture, Guangdong Provincial Key Laboratory for the Development Biology and Environmental Adaptation of Agricultural Organisms, College of Life Sciences, South China Agricultural University, Guangzhou 510642, China; Department of Chromosome Biology, Max Planck Institute for Plant Breeding Research, Carl-von-Linné-Weg 10, Cologne 50829, Germany; Department of Chromosome Biology, Max Planck Institute for Plant Breeding Research, Carl-von-Linné-Weg 10, Cologne 50829, Germany; School of Automation Science and Engineering, Faculty of Electronic and Information Engineering, Xi’an Jiaotong University, Xi’an 710049, China; State Key Laboratory of Crop Gene Resources and Breeding, Key Laboratory of Soybean Biology (Beijing), Ministry of Agriculture and Rural Affairs, Institute of Crop Science, Chinese Academy of Agricultural Sciences, Beijing 100081, China; Beijing Key Laboratory of Plant Gene Resources and Biotechnology for Carbon Reduction and Environmental Improvement, and College of Life Sciences, Capital Normal University, Beijing 100048, China; Beijing Key Laboratory of Plant Gene Resources and Biotechnology for Carbon Reduction and Environmental Improvement, and College of Life Sciences, Capital Normal University, Beijing 100048, China; Beijing Key Laboratory of Plant Gene Resources and Biotechnology for Carbon Reduction and Environmental Improvement, and College of Life Sciences, Capital Normal University, Beijing 100048, China; Beijing Key Laboratory of Plant Gene Resources and Biotechnology for Carbon Reduction and Environmental Improvement, and College of Life Sciences, Capital Normal University, Beijing 100048, China; Beijing Key Laboratory of Plant Gene Resources and Biotechnology for Carbon Reduction and Environmental Improvement, and College of Life Sciences, Capital Normal University, Beijing 100048, China; Beijing Key Laboratory of Plant Gene Resources and Biotechnology for Carbon Reduction and Environmental Improvement, and College of Life Sciences, Capital Normal University, Beijing 100048, China; Beijing Key Laboratory of Plant Gene Resources and Biotechnology for Carbon Reduction and Environmental Improvement, and College of Life Sciences, Capital Normal University, Beijing 100048, China; Beijing Key Laboratory of Plant Gene Resources and Biotechnology for Carbon Reduction and Environmental Improvement, and College of Life Sciences, Capital Normal University, Beijing 100048, China; State Key Laboratory of Crop Gene Resources and Breeding, Key Laboratory of Soybean Biology (Beijing), Ministry of Agriculture and Rural Affairs, Institute of Crop Science, Chinese Academy of Agricultural Sciences, Beijing 100081, China; Beijing Key Laboratory of Plant Gene Resources and Biotechnology for Carbon Reduction and Environmental Improvement, and College of Life Sciences, Capital Normal University, Beijing 100048, China

## Abstract

In soybean (*Glycine max* (L) Merr.), the circadian clock orchestrates rhythmic molecular, metabolic, and physiological processes that determine yield potential and restrict cultivars to a narrow latitudinal range. However, the role of dynamic chromatin states in rhythmic transcription remains elusive. Here, we integrated time-series assay for transposase-accessible chromatin using sequencing, chromatin immunoprecipitation sequencing, and RNA-seq to map the genome-wide circadian chromatin accessibility landscape under free-running conditions in soybean. We identified 11 core co-oscillation groups (CCOGs), whose chromatin accessibility was synchronized with mRNA oscillations. Accessible chromatin regions (ACRs) within these CCOGs are enriched for binding motifs of the core circadian oscillators, which regulate the expression of their target genes. Two functionally distinct ACRs, which respectively regulate the circadian rhythmicity and stable expression of *LATE ELONGATED HYPOCOTYL 1a* (*GmLHY1a*), were characterized. Natural variants of these ACRs were correlated with the latitudinal adaptation of soybeans. In addition, mutations in core oscillator components, including *GmLHYs* and *LUX ARRHYTHMO* (*GmLUXs*), disrupt chromatin oscillation in CCOGs. For instance, altered chromatin accessibility was detected at key binding sites, such as the region where GmLHY1a interacts with the ACR associated with *GmPIF4* (*PHYTOCHROME INTERACTING FACTOR 4*). Collectively, these findings reveal a mechanism wherein chromatin accessibility rhythms orchestrate genome-scale transcriptional programs and provide a large-scale, time-resolved, multi-omics resource to facilitate crop breeding for improved environmental adaptation.

## Teaser

Circadian changes in chromatin accessibility—how loosely or tightly DNA is packaged—temporally regulate the expression levels of rhythmic genes in soybeans, helping plants adapt to their environment.

## Introduction

Organisms have evolved endogenous circadian clock to adapt to environmental changes induced by Earth's rotation (Wijnen and Young 2006). This internal timekeeping mechanism rhythmically regulates various physiological processes. In the model plant *Arabidopsis thaliana*, more than 30% of genes maintain diurnal rhythms, even under free-running conditions (Bendix et al. 2015). Given the crucial role of circadian clock in plant growth, development, and environmental adaptation, it is essential to maintain precise, autonomous circadian rhythms within cells (Dodd et al. 2005; Xu et al. 2022). In plants, the core oscillators of the circadian clock include CIRCADIAN CLOCK ASSOCIATED 1 (CCA1), LHY, TIMING OF CAB EXPRESSION 1 (TOC1), PSEUDO-RESPONSE REGULATOR (PRR), LUX, and EARLY FLOWERING (ELF), which are expressed sequentially throughout the day and night, maintained by conserved transcription-translational feedback loops (TTFLs) (Hazen et al. 2005; Wijnen and Young 2006; Millar 2016). Histone modifications play a critical role in regulating the expression of these core oscillators. For example, SET DOMAIN GROUP 2 (AtSDG2, a histone methyltransferase) and Jumonji C (JmjC) domain-containing protein 14 (AtJMJ14, a histone demethylase) regulate the expression balance of *AtCCA1* and *AtLHY* by writing and removing H3K4me3, respectively, in *Arabidopsis* (Song et al. 2019). In mammals, HISTONE DEACETYLASES3 (HDAC3) and LYSINE SPECIFIC DEMETHYLASE 1 (LSD1) regulate the expression of core oscillators like *BASIC HELIX-LOOP-HELIX ARNT LIKE 1* (*BMAL1*, a core clock component in mammals) and *PERIOD CIRCADIAN REGULATOR 2* (*PER2*, a core clock component in mammals) by modulating histone modifications (Alenghat et al. 2008; Nam et al. 2014). However, the mechanism underlying global rhythmic transcription remains unclear.

Chromatin states, influenced by regulatory proteins such as chromatin remodeling factors, are thought to involve nucleosome depletion or dynamic nucleosome modification or displacement (Lorch et al. 1987; Lorch and Kornberg 2017; Chen et al. 2022). ACRs are enriched with cis-regulatory elements (CREs) in eukaryotic genomes (Gross and Garrard 1988; Park et al. 2018). Loosened chromatin enhances the binding of transcription factors to target DNA sequences, further facilitating the recruitment of RNA Polymerase II (RNAPII) to modulate transcription (Park et al. 2018). Recent studies have identified several critical CREs as enhancers marked by functional epigenetic ACRs in *Arabidopsis* and crops (Adrian et al. 2010; Liu et al. 2014; Hendelman et al. 2021; Tian et al. 2021; Aguirre et al. 2023). However, the dynamics of ACR-mediated gene regulation, especially in circadian contexts, are poorly understood.

This gap is particularly evident in crops, where investigating the regulatory mechanisms of circadian clocks remains challenging due to the genomic complexity (Li et al. 2019). To bridge this knowledge gap, we generated a high-quality multi-omics landscape in soybean (*Glycine max* (L) Merr.) under free-running conditions by integrating the ATAC-seq, ChIP-seq and RNA-seq time-course data. Our work revealed a strong synchronization between circadian transcription and chromatin states, 11 typical co-oscillation clusters, and a rhythmic ACRs-GmLHY-targets regulatory axis. This epigenetics-dependent cis-element landscape may offer a powerful approach to decipher the circadian regulatory network and facilitate precision breeding in soybean.

## Results

### Integrative multi-omics analysis reveals co-oscillations of chromatin accessibility and gene transcription in soybean

**Figure 1:**
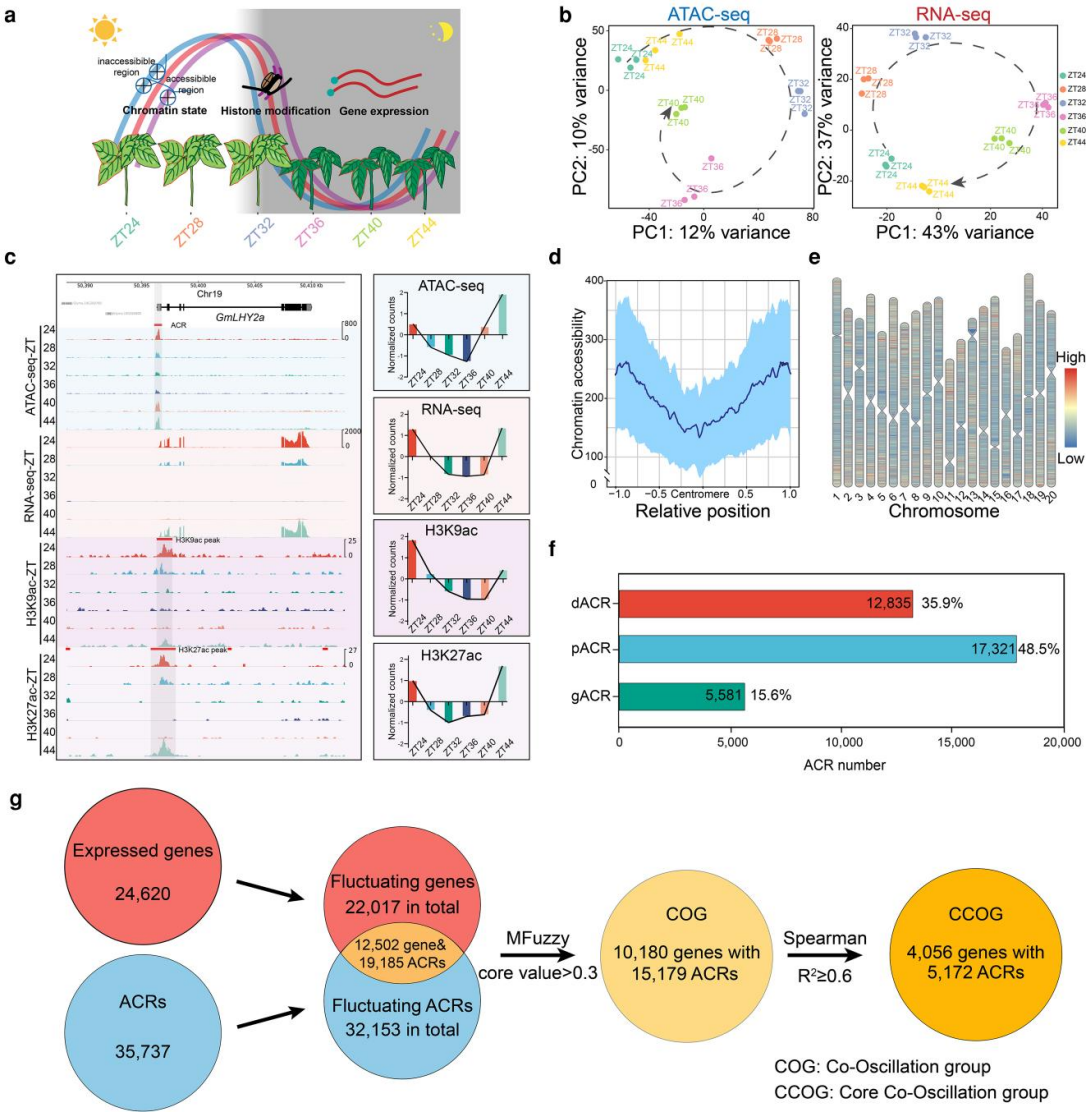
Screening co-oscillation genes based on the synchronization of chromatin accessibility and gene transcription in soybeans. a) Overview of the experimental design. Williams 82 grew under short day (SD) condition (8 hL:16 hD) for 18 d and transferred to constant light (LL) condition 24 h and harvested at 6 designed time points (ZT24, ZT28, ZT32, ZT36, ZT40, ZT44) during the LL period. b) PCA for ATAC-seq (left) and RNA-seq (right). c) ATAC-seq, RNA-seq, and ChIP-seq tracks at the *LHY2a* locus. Differentially accessible chromatin peaks and histone modification regions are shadowed. Normalized Z-scores for each assay across time points are shown on the right. d) Relative distance from the centromere is plotted on the *x* axis. The *y* axis represents chromatin accessibility. The dark line shows the mean chromatin accessibility at each position, and the light area indicates the standard deviation (sd). Each bin is 20 kb. e) Red indicates high chromatin accessibility, while blue indicates low accessibility. Each bin is 20 kb, and the central gap represents the centromere region. f) Genome-wide distribution of ACR categorized as distal ACR (dACR), proximal ACR (pACR), and genic ACR (gACR) are shown. dACR: ACRs are located more than 1 kb away from the TSS or TES of a gene; pACRs fall within 1 kb of a gene's TSS and TES; are locatedCRs located within the gene body, including regions within 5′ UTR and 3′UTR. The number of ACRs in each category is indicated in the bar plots. The percentages relative to the total ACRs are shown on the right. g) Proportion of gene expression and chromatin accessibility regulated by temporal order. Genome-wide analysis identified 24,620 expressed genes and 35,737 ACRs. Among these, 22,017 fluctuating genes and 32,153 fluctuating ACRs were screened based on standard deviation of their expression or accessibility profiles, respectively. Of the 22,017 fluctuating genes, 12,502 harbored at least 1 fluctuating ACR in their vicinity or within the gene body. Subsequently, the correlation between fluctuating ACRs and their associated fluctuating genes was analyzed using the *Mfuzzy* package. Those with a core correlation value > 0.3 were defined as COG, encompassing 10,180 genes and 15,179 ACRs. Furthermore, genes with highly co-oscillating gene expression levels and chromatin accessibility were screened using Spearman correlation coefficient. Finally, a total of 4,056 genes and their corresponding 5,172 ACRs were identified, which were designated as CCOG.

To investigate the circadian oscillation of epigenetic modification and gene expression in soybean, we generated ATAC-seq (119.7 Gb), RNA-seq (103.0 Gb), and ChIP-seq (90.6 Gb) using antibodies against H3K9ac and H3K27ac in wild-type Williams 82 (Ws82; Table S1). Specifically, leaf samples were collected at 6 time points every 4 h over a day period under free-running conditions (Fig. 1a; Materials and Methods). For a more comprehensive assessment of the data quality, principal component analysis (PCA) was conducted on both ATAC-seq and RNA-seq data (Fig. 1b; Materials and Methods). The results demonstrated robust correlations among replicates and conspicuous differences among distinct ZT samples in addition to presenting a temporal progression. Q30 SPOT values ranging from 34.0% to 58.6% further validate the high sequencing quality of the dataset (Fig. S1a). Using a customized pipeline, ACRs were first identified independently for each of the 6 time points. After removing redundant regions, a total of 35,737 ACRs were finally obtained through merging (Lu et al. 2019; Wang et al. 2021) (Fig. S1b).

We found that the chromatin accessibility exhibits synchronized oscillations along with enrichments of H3K9ac and H3K27ac, and gene expression, all exhibiting a clear 24-h oscillation pattern (Fig. 1c). Furthermore, genome-wide analysis showed that assessed dynamic chromatin changes are most pronounced on the chromosome arms (Fig. 1d, e), which is consistent with active transcription regions (Wang et al. 2021). These results suggest that chromatin accessibility may be synchronized with gene expression on a genome-wide scale. To investigate this further, we analyzed the distribution of ACRs across the genome. It was found that 5,581 (15.6%) of the ACRs are located in the genebody (gACRs, defined as located in the gene's exon or intron, including 5′ and 3′UTR) and the majority of ACRs (17,321 ∼48.5%) were located in the proximal region (pACR, defined as within 1 kilobase (kb) of transcription start site (TSS) or transcription end site (TES). We also found that 12,835 ACRs (35.9%) were located more than 1 kb from the TSS or TES, which are defined as distal ACRs (dACRs) (Fig. 1f) (Ricci et al. 2019). Overall, ACRs are widely distributed across the genome, with the highest enrichment in proximal regions and moderate enrichment in distal regions.

To investigate chromatin accessibility oscillations, 35,737 ACRs and 24,620 expressed genes were analyzed (Fig. S1c and d; Table S2 and S3). We identified 21,153 fluctuating ACRs (59.5%) and 22,017 fluctuating genes (90.8%) in total, which were defined by a chromatin accessibility or expression level coefficient of variation (CV) greater than 10 percentiles (see Materials and Methods). For genes exhibiting stable expression, the underlying reasons could be multi-fold. For instance, the criteria for identifying differential expressions are relatively stringent; cell-type-specific changes may not be effectively captured at the leaf tissue level; and post-transcriptional regulatory mechanisms may be involved, among others. By overlapping fluctuating genes and fluctuating ACRs, we found that 19,185 fluctuating ACRs (53.7%) were associated with the corresponding 12,502 fluctuating genes (50.8%), with each of these genes harboring at least 1 fluctuating ACR in its genomic vicinity. Furthermore, to identify co-oscillating ACRs and gene expression levels, we calculated the correlation between chromatin accessibility and gene expression profiles using R package *Mfuzz* (Kumar and Futschi 2007) (see Materials and Methods). This analysis identified 10,180 oscillating genes (41.3%) and 15,179 oscillating ACRs (42.5%), collectively referred to as the COG (co-oscillation group). The ratio of oscillating genes is similar to *Arabidopsis* (Michael et al. 2008). Furthermore, to identify the high correlation between gene expression and chromatin accessibility, we defined synchronous oscillation genes as those with a high correlation (R^2^ ≥ 0.6, using Spearman rank correlation coefficient) between gene expression and chromatin accessibility. This identified 4,056 synchronous genes (16.5%) and their associated 5,172 ACRs (14.5%), which we termed the core co-oscillation group (CCOG) (Fig. 1g; Table S4). We further investigated the distribution of chromatin accessibility in gene loci, and the accessible regions are mostly enriched around the TSS and TTS (Fig. S2).

In summary, our ATAC-seq, ChIP-seq, and RNA-seq profiling reveals a dynamic landscape of epi-genome and transcriptome in soybean, highlighting a general oscillatory pattern of chromatin accessibility across the genome.

### Eleven co-oscillation clusters were characterized based on the oscillating patterns of ACRs and gene expressions

**Figure 2:**
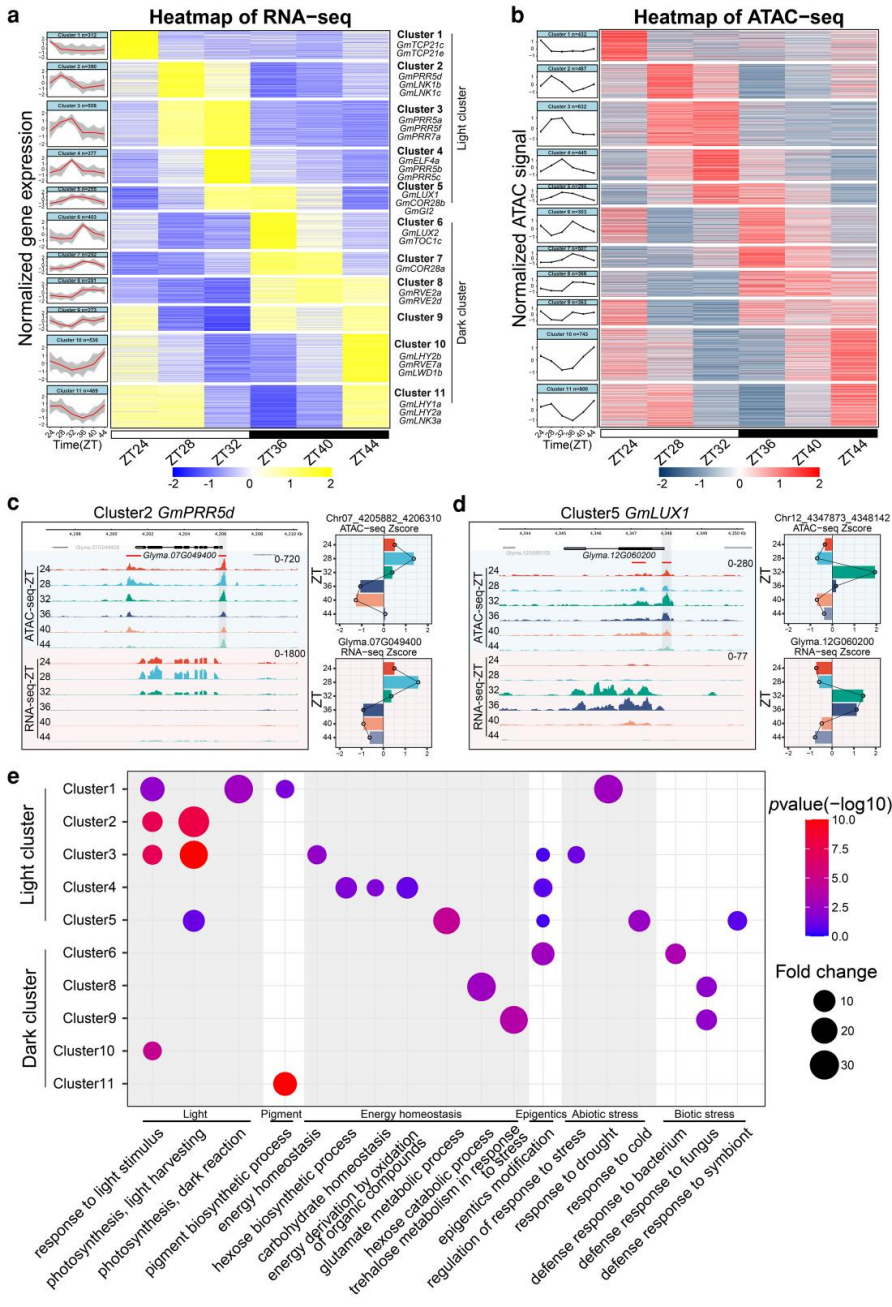
Co-oscillation genes were clustered rhythmically and pathway-enriched in sequential. a) Cluster analysis of 4,056 genes clustering by fuzzy c-means cluster approach. The line represents the mean gene expression level in each cluster. The shaded region shows the expression level of individual in each cluster. The heatmap shows the Z-score of expression for each gene grouped by different clusters. b) The line plot showing 11 clusters included 5,172 chromatin accessible regions, the error bar shows 95% confidence interval. The heatmap shows the Z-score of chromatin accessible level for each region grouped by different clusters. c, d) ATAC-seq and RNA-seq genome tracks for *PRR5d* and *LUX1* are shown on the left. The right panels display quantified chromatin accessibility of the shaded ACRs following Z-score normalization, with colors matching the 6 ZT time points in the genome tracks. e) GO enrichment analysis of identified clusters. N for Clusters 1–11 is 312, 390, 508, 377, 255, 403, 252, 281, 273, 536, and 469, respectively. The *P* values were calculated by Fisher exact test.

To explore the patterns of the oscillation genes, clustering analysis was performed, which revealed 11 distinct co-oscillation clusters based on temporal patterns of gene expression and chromatin accessibility (Fig. 2a, b; Materials and Methods). Cluster 1 to 5 exhibited peaks in both gene expression and chromatin accessibility during the subjective light period, defined as light clusters, while clusters 6 to 11 showed peaks during the subjective dark, defined as dark clusters (Fig. 2a). Each cluster displayed a characteristic expression pattern, which coincided with the expression of core clock genes such as morning loop member *GmPRR5d* in cluster 2 and evening complex member *GmLUX1* in cluster 5 (Fig. 2c, d). Notably, light clusters genes included *GmPRR5b*, while dark clusters genes included *GmTOC1c* and circadian regulated genes, like *GmCOR28b* and *GmRVE2d* (Fig. S3a–d) (Li et al. 2016; Gray et al. 2017).

In the CCOG, approximately 52.0 to 65.8% of ACRs in each cluster were located in proximal regions, accounting for a higher proportion than genome-wide (Fig. S3e and f). This suggests that putative CRE within these ACRs may regulate gene oscillation. Previous studies have shown that histone acetylation marks active CRE (Andersson and Sandelin 2020). We found that 46.6% and 25.6% CCOG ACRs were enriched in H3K9ac and H3K27ac modifications, respectively, with a high correlation to gene expression (R^2^ ≥ 0.6, using Spearman rank correlation coefficient) (Fig. S4a and b; Table S5). Remarkably, the summits of H3K9ac and H3K27ac modifications were located downstream of ACRs, on average ∼250 bp away (Fig. S4c and d).

Subsequently, we investigated the pathways associated with the light and dark clusters. Gene ontology (GO) analysis revealed that cluster 1 to 3, which are activated in the early-morning, were significantly enriched with genes in pathways related to photosynthesis and light signal transduction, including photoreceptors such as *GmCRY1b-1* and *GmCRY2b*, as well as *CHLOROPLAST A/B BINDING PROTEIN* (*GmCAB*) (Fig. 2e). Upon exposure to sunlight, plants initiate light signal transduction, photosynthesis and carbon fixation. Clusters 4 to 5 were enriched with genes in pathways related to light harvesting, carbon and nitrogen metabolism, and response to cold (abiotic stresses). As light intensity decreased, plants exhibited enhanced carbon-nitrogen metabolic coupling and heightened responsiveness to cold stress. Notably, the dark clusters (clusters 6 to 9) were enriched with genes involved in pathways related to hexose catabolic process, trehalose metabolism–a pathway associated with response to abiotic and biotic stresses, while cluster 10 to 11 were involved in photosynthesis and pigment biosynthetic processes (Fig. 2e). Following sunset, plants experience heightened abiotic and biotic stresses and activate relative response pathways to avoid damage. And at dawn, they initiate the pigment biosynthesis in preparation for the photosynthesis of the following day (Fig. 2e). We hypothesize that chromatin accessibility can regulate gene expression to promote the balance between plant development and stress response.

Since ACRs on target genes are frequently bound by transcription factors, we identified motifs within ACRs to analyze the transcriptional regulatory network. To construct the core regulatory networks, we performed motif enrichment analysis for each clock gene to identify upstream regulators (*P* < 0.005; Materials and Methods). This analysis revealed a network containing 38 clock genes and 542 regulatory interactions between TFs and targets (Fig. S5; Table S6). On average, each ACR interacts with 14 clock genes. The transcriptional regulatory network also captured well-known interactions in *Arabidopsis*, such as those between *LHYs* and *PRRs*, between *LHY* and *TOC1 s*, between *LUXs* and *PRR* (Wijnen and Young 2006; Nohales and Kay 2016).

In summary, we identified oscillating cluster genes with time-sequential peaks in gene expression and chromatin accessibility, indicating well synchronized regulation of clock-controlled gene transcription and chromatin dynamic across the genome. The 11 clusters were enriched in typical biological processes in temporal order. This result indicates that chromatin accessibility is a critical prerequisite for gene circadian expression, aiming to balance the development and the response to endogenous cues and environmental stresses.

### Two ACRs exhibit distinct roles in regulating circadian rhythmic and stable expression of *GmLHY1a*

**Figure 3:**
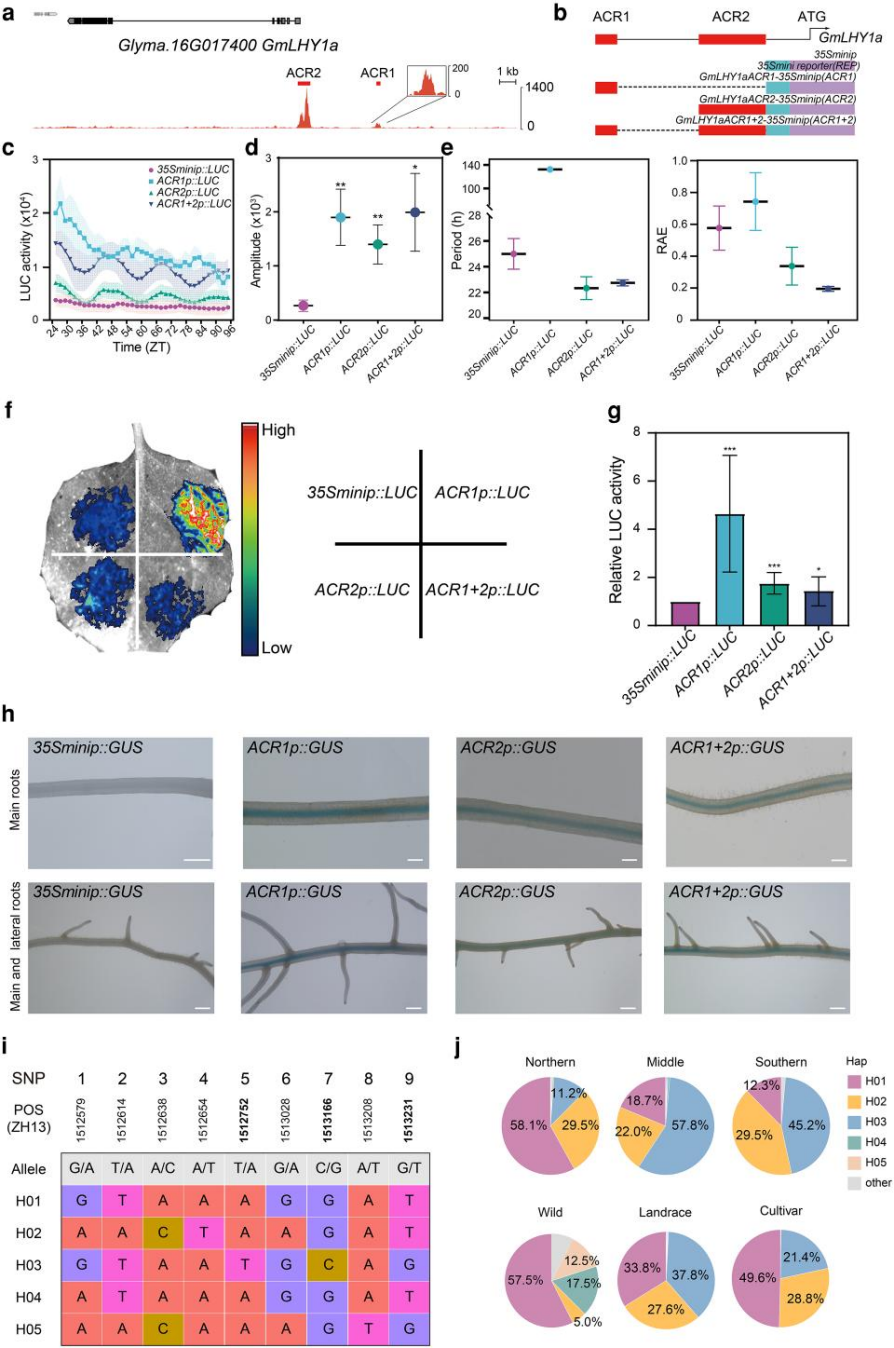
The oscillating pattern of *GmLHY1* transcription was regulated by 2 distinct functional ACRs. a) Genome browser view of chromatin accessibility *GmLHY1a* locus. The red boxes represent ACR1 and 2. b) A diagram showing the relative positions of the 2 ACRs derived from *GmLHY1a* locus. c) Comparative luminescence analyses of *GmLHY1a ACRp::LUC* rhythms in hairy root. The hairy roots carrying the LUC reporter were grown under 16hL:8hD cycles for 14 to 21 d before the constant dark entrainment. d, e) Period length and relative amplitude were calculated by FFT-NLLS analysis according to data from ZT24 to ZT96 (2 to 4 replicates per genotype). The period, amplitude, and relative amplitude error (RAE) are used to evaluate the strength of rhythmicity. Data represent mean ± sd. A Student *t* test was used to generate the *P* values, *P < 0.05, **P < 0.01. f, g) *LUC* reporter gene was driven by different *GmLHY1a ACRs* assayed in *Nicotiana* leaves. The relative LUC signal derived by *GmLHY1a ACR*. The signal was normalized by *35Sminip::LUC*. Data represent mean ± sd. A Student *t* test was used to generate the *P* values, *P < 0.05, ***P < 0.001. h) Soybean transgenic roots showing GUS signals. The *GmLHY1a ACRs* construct used in transformation is marked on each seedling image. The bar indicates 1 mm for all images. i) The haplotypes of *GmLHY1a ACR2*, with ZH13 accession as reference genome (1979 accessions in total). The SNPs within the ACR2 of *GmLHY1a* were extracted using *VCFtools* (0.1.17). j) Upper: Proportion of accessions (1979 accessions in total) with homozygous haplotypes in 3 geographic regions of China: north of the Yellow River (Northern), between the Yellow and Yangtze Rivers (Middle), and south of the Yangtze River (Southern). Bottom: Proportion of the accessions (40 from wild accession, 1,125 from cultivar accession, 532 from landrace accession) with homologous haplotypes in each distinct geography region and germplasm groups.

We identified 2 ACRs located upstream of the *GmLHYs* loci that are conserved across 4 homologous *GmLHYs* genes in soybean and exhibit a similar positional relationship with respect to each of these paralogs (Fig. S6). The typical *GmLHY1a* with the distal *ACR1* spanning from −5,753 to −5,518 bp upstream of ATG, and the proximal *ACR2*, ranging from −1,654 to −924 bp (Fig. 3a). *GmLHY1a* is highly expressed at ZT24 and ZT44 when the proximal ACR2 chromatin are more accessible while the distal *ACR1* does not show oscillatory patterns. Other *GmLHYs* homologous genes exhibit both comparable expression patterns and highly similar ACR localization profiles. Recent studies in both animals and plants have highlighted the significant role of distal CRE in regulating gene transcription, like *AtFT block* C enhancer and *AtGI* CRM2 (Adrian et al. 2010; Berns et al. 2014; Zhu et al. 2015). However, how ACRs function as CRE to regulate *LHY* as well as other core oscillator genes' expression remains unclear in plants. To address this, we aimed to dissect and validate the function of these 2 ACRs in the *GmLHY1a* promoter.

Both *GmLHY1a ACR1* and *ACR2* were cloned from Ws82 genomic DNA and inserted into a reporter vector containing a *35S minimal* promoter (−46 to +1 bp, *35S mini*), which is essential for transcriptional initiation with no (or low) basal expression (Amack and Antunes 2020). The constructs were then transformed into Ws82 hairy roots and assayed for LUC expression in free-running conditions following entrainment (Fig. 3b). We found that the LUC signal driven by *35S mini* alone was almost undetectable in the hairy roots (Fig. 3c). When only *ACR2* was present, it showed high diurnal circadian rhythmic expression, although with a lower amplitude compared with earlier studies (Dong et al. 2021). In contrast, when only *ACR1* was included, the signal was high but lacked a clear diurnal rhythm (Fig. 3c–e). However, when both *ACR1* and *ACR2* were placed upstream of *35S mini*, the resulting LUC signal exhibited high-amplitude 24-h rhythmic gene expression.

Taken together, these results suggest that the *ACR2* functions as a transcriptional regulatory cis-element, controlling the rhythmic expression of *GmLHY1a*, while the distal *ACR1* appears to have enhancer activity, activating the overall expression of *GmLHY1a*, suggesting the distinct roles of *ACR1* and *ACR2* regulate *GmLHY1a* expression level and rhythm respectively.

To further validate the enhancer activity of *ACR1*, we performed a transient assay in *Nicotiana*. Notably, compared with*35S mini* control*, ACR2* driven higher LUC signals were observed in *Nicotiana* leaves (Fig. 3f, g). In soybean hairy root, we transformed into a *GUS* reporter driven by the same ACRs fused with the *35S mini* promoter. GUS assay in hairy roots was consistent with LUC assay, confirming that *ACR1* functions as an enhancer at the *GmLHY1a* locus (Fig. 3h).

Some known circadian clock genes such as GmPRR3a/3b can bind to the promoter region to regulate the expression of *GmLHY1a* (Lu et al. 2020). Our findings show that their binding site is located within the *ACR2* region exactly. To explore potential circadian clock regulators, we performed motif enrichment analysis to screen for additional transcription factors capable of binding to the *ACR2* region (Fig. S7a). Further, experimental validation identified transcription factors such as GmFBH3 and GmSPT, which were predicted to bind *ACR2*, act as regulators of clock gene expression, and their functions were confirmed through dual luciferase (dual LUC) assay (Fig. S7b). Our findings indicate that ACRs play distinct roles in the *GmLHY1a* transcriptional regulation.

To understand how soybeans evolved and domesticated to adapt, we examined the genome variations in the *GmLHY1a ACR2* of 1979 previously re-sequenced soybean accessions (Zhou et al. 2015b; Liu et al. 2020). We identified 5 reliable haplotypes, H01-H05, in *GmLHY1a ACR2* (Fig. 3i). Interestingly, as latitude increases, the homozygosity rate of SNP 5, 7 and 9 exhibits a distinct upward trend, indicating a significant positive correlation (Fig. S8a–c). After domestication and as soybean adapted to different latitudes, SNP 5, 7 and 9 were strongly selected in accessions from various regions (Fig. 3j). We next examined how the distributions of the major H01-H05 alleles within the subset of Chinese accessions differed according to their geographic origins (Fig. 3j). As observed for the entire panel, H01 was strongly enriched in the northern region and fixed in cultivars. Within both landraces and improved cultivars, H01, H02 and H03 alleles occupy a dominant position (Fig. 3j). By analyzing published transcriptome data (Liu et al. 2020; Zhang et al. 2022a), we found that H01 exhibited the highest *GmLHY1a* expression, whereas accessions carrying H03 showed the lowest expression (Fig. S8d). Combined with the above geographic distribution data, we found that the SNPs within this *ACR2* regulate *GmLHY1a* expression and shape the geographic distribution of accessions. This conclusion corroborates that *GmLHY1a* contributes to the adaptation of soybean from its temperate origin to tropical regions (Dong et al. 2021). We collected the EMS-induced point mutation in *ACR2* (m1 and m2) from iSoybean database (Zhang et al. 2022a) (Fig. S8e). Sampling was performed at ZT24 and ZT36, and *GmLHY1a* expression levels were measured via RT-qPCR (Fig. S8e). We found that compared with WT plants, the *GmLHY1a* expression levels in both mutant materials were significantly downregulated at both time points.

Taken together, the distal *ACR1*, acts as a general enhancer in transient assays, increasing the baseline expression of a reporter gene driven by the *35S mini*. In contrast, the proximal *ACR2* functions as an essential core cis-element to induce transcription in response to the circadian clock. Strong correlations between SNP1 and SNP3 and latitude suggest that *ACR2* variants play a critical role in supporting the geographic distribution of soybeans.

### ACRs from CCOG and their enriched pathways are regulated by core clock genes

To investigate the role of core clock genes in controlling chromatin accessibility in soybean, we profiled chromatin accessible regions using ATAC-seq in the WT and CRISPR/Cas9-edited mutants deficient in circadian clock genes *GmLHY*s (*Gmlhy1a/1b/2a/2b*) and *GmLUXs* (*Gmlux1/2*) under continuous light condition (Cheng et al. 2019; Bu et al. 2021), with 2 independent replicates per time point, demonstrating robust correlations among replicates and conspicuous differences among distinct ZT samples (Fig. S9a). A similar assay was performed to detect chromatin accessibility changes at ZT24 and ZT36, representing subjective light and dark condition respectively. ZT24 corresponds to the peak of *GmLHYs* expression, while ZT36 corresponds to the peak of *GmLUX*. The chromatin accessibility landscape of *GmLHY2a* in WT was similar to that shown in Fig. 1, with the peak phase of chromatin accessibility concentrated at dawn (Fig. S9b).

**Figure 4:**
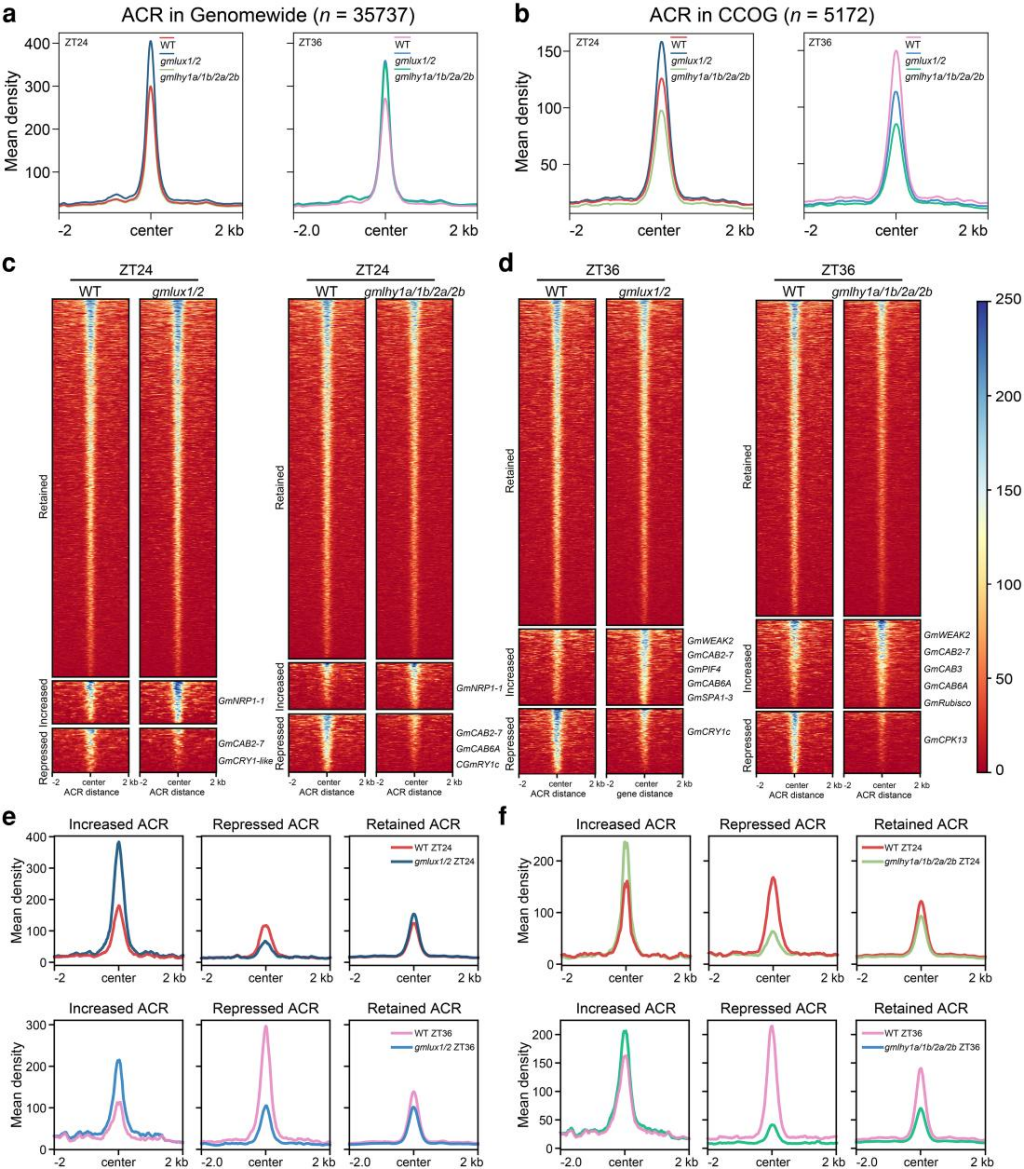
ACRs from CCOG are regulated by core clock genes. a) Average distribution of WT and mutant's chromatin accessibility signals in genome wide. Read counts per gene were summed in 50-bp bins. Left, ZT24. Right, ZT36. b) Average distribution of WT and mutant's chromatin accessibility signals in CCOG. Read counts per gene were summed in 50-bp bins. Left, ZT24. Right, ZT36. c, d) Heatmap and metagene plots reflecting the ATAC-seq signals of CCOG over the retained, induced, or repressed chromatin accessibility sites of *Gmlux1/2* and *Gmlhy1a/1b/2a/2b* in WT at ZT24 and ZT36. e, f) Genome track visualization of ATAC-seq profiles showing chromatin accessibility at the *GmCRY1-like* and *GmCAB2-7* locus of WT and mutants (ZT24, ZT36) under free-running conditions.

Significant differences were observed between WT and mutants at genome scale, suggesting that chromatin accessibility across the genome was significantly altered in *Gmlhy1a/1b/2a/2b* and *Gmlux1/2* mutants compared with WT (Fig. 4a). No significant changes in average chromatin accessibility were observed across the 6 time points in WT (Fig. S10). At ZT24, chromatin accessibility in *Gmlux1/2* was 33% higher than in WT, while at ZT36, chromatin accessibility in *Gmlhy1a/1b/2a/2b* and *Gmlux1/2* was 25% higher than in WT (Fig. 4a). In the CCOG, differences in chromatin accessibility between WT and mutants were even more pronounced. At ZT24, chromatin accessibility in *Gmlux1/2* was higher than in Ws82, while *Gmlhy1a/1b/2a/2b* had the lowest accessibility. At ZT36, WT had the highest chromatin accessibility, followed by *Gmlux1/2*, with *Gmlhy1a/1b/2a/2b* still showing the lowest accessibility (Fig. 4b). These results indicate that the core clock genes regulate the circadian clock through not only transcription regulation but also via chromatin accessibility, especially those genes expression circadian rhythms. And *Gmlux* mutants cause more significant changes of chromatin status in several dark clusters 6 to 8 (Fig. S11). The most significant differences in chromatin accessibility among mutants occur at time points when *GmLUXs* (ZT24) or *GmLHYs* (ZT36) expression is lowest in WT plants. We measured the expression levels of *GmLHY1a* and *GmLUX1* in *Gmlux1/2* and *Gmlhy1a/1b/2a/2b* mutants and observed shifts in their expression rhythms (Fig. S12). The core clock genes may play a critical role in maintaining chromatin status oscillation.

To explore how the chromatin accessibility is determined by core clock components, we identified differentially enriched ACRs between mutants and WT (Fig. 4c, d; Table S8). Any ACR in CCOG was considered as significant if it was identified in both biological replicates using *DESeq2* (*P* < 0.05) and showed increased or repressed tagmentation signals >1.5-fold. These differential ACRs associated genes included *GmNRP1-1*, *GmCAB2-7*, *GmCRY1c*, *GmCRY1-like*, *GmCAB6A*, *GmWEAK2*, *GmPIF4*, *GmSPA1-3*, *GmRUBISCO*, and *GmCPK13* (Zhou et al. 2015a; Zhu et al. 2016; Wei et al. 2020). These genes are enriched in pathways that are similar to those identified in CCOG, as revealed by GO analysis (Fig. S13). Among these genes, *GmCAB2-7* and *GmCRY1-like*, which is response to light signaling, light capture, and energy transfer (Aanderson and Kay 1995 ; He et al. 2022), the chromatin accessibility is moderately changed in *Gmlux1/2* and *Gmlhy1a/1b/2a/2b* mutants (Fig.4e, f; Fig. S14a and b). We also measured the expression levels of *GmCAB2-7* and *GmCRY1-like* in the mutants and found that their expression patterns were significantly altered (Fig. S14c and d).

We observed that ACRs are associated with the regulation of core clock gene expression, while the rhythmic activity of core clock genes in turn correlates with dynamic changes in downstream ACR accessibility. This points to a coordinated, reciprocal interaction between chromatin accessibility and circadian components, rather than a strict hierarchical regulatory pattern. Specifically, mutations in core clock components disrupt this regulatory cascade: compromised clock gene function leads to altered chromatin accessibility at downstream target genes involved in circadian-linked fitness pathways. This supports a feedback loop where ACRs may contribute to modulating core clock gene expression, and clock gene products reciprocally impact rhythmic chromatin remodeling and downstream transcriptional outputs. This bidirectional interplay is consistent with a role in maintaining circadian homeostasis, though further studies—such as assessing circadian rhythmicity of key physiological processes (eg, photosynthesis, flowering) in lines with perturbed ACRs-clock interactions—would be needed to confirm its relevance to circadian fitness.

### GmLHY1a directly targets and regulates *GmPIF4* through ACRs

**Figure 5:**
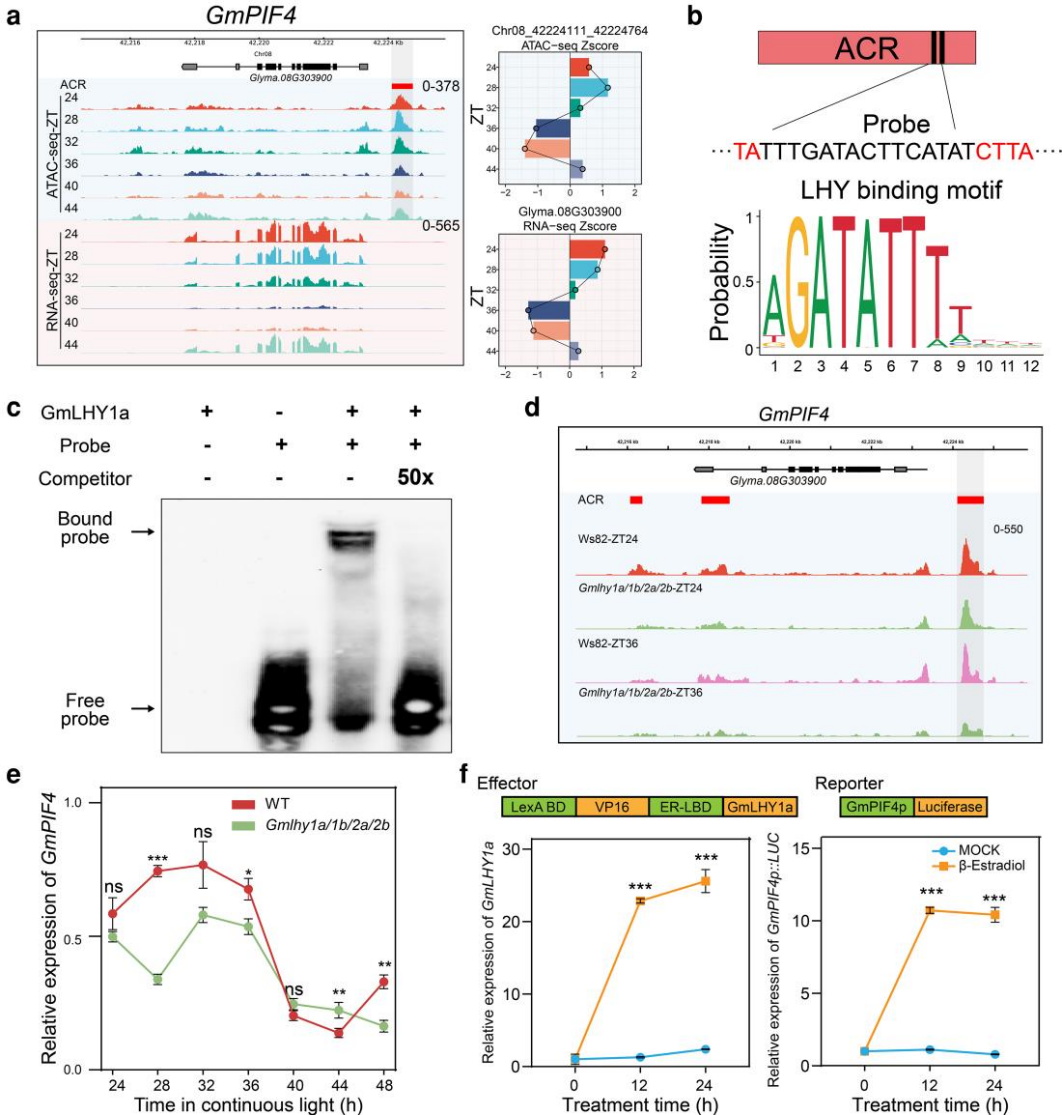
GmLHY1a regulates *GmPIF4* through associating with motifs at ACRs. a) Genome browser track view of ATAC-seq profiles of chromatin accessibility and RNA-seq data at *GmPIF4* locus at 6 time points (ZT24, ZT28, ZT32, ZT36, ZT40, ZT44) under free-running conditions. The red box represents the ACR. b) The GmLHY1a protein-binding sites within the ACR of the Gm*PIF4* promoter, along with the GmLHY1 binding motif, are shown. c) EMSA analysis demonstrated that the GmLHY1a protein binds to the probe derived from the *GmPIF4* ACR. Specifically, recombinant purified His-tagged GmLHY1a protein showed binding to the biotin-labeled probes tested. Unlabeled probes were used as competitors in the assay. d) Genome track visualization of ATAC-seq profiles showing chromatin accessibility at the *GmPIF4* locus of WT and mutants across 6 time points (ZT24, ZT28, ZT32, ZT36, ZT40, ZT44) under free-running conditions. The red box represents the ACR. e) The circadian rhythm of *GmPIF4* expression was measured in WT and *Gmlhy1a/1b/2a/2b* knockout mutants via RT-qPCR, *GmACTIN* used as control. Soybean seedlings were first grown under SD conditions at 24 °C for 19 d, after which they were transferred to continuous light. One-way ANOVA-test was used to generate the *P* values, *P  *<* 0.05, **P < 0.01, ***P < 0.001. Abbreviation: ns, nonsignificant. f) Upper: Constructs used for the transient transfection assay. LexA BD, LexA DNA-Binding Domain, VP16, transcription activity domain, ER-LBD estrogen receptor ligand-binding domain. The reporter vector is *GmPIF4* promoter (2,387 bp) derived LUC. Bottom: results from 3 independent replications; the value of each replication is represented by a dot. A Student *t* test was used to generate the *P* values, ***P < 0.001.

We explored the associations between the core clock genes and ACRs. For example, *GmPIF4* is a critical regulator of photomorphogenic and thermomorphogenic growth (Huq and Quail 2002; Vinod Kumar et al., 2012). We found that *GmPIF4* is associated with 3 ACRs, of which 1 is located in the upstream of ATG and oscillates significantly with gene expression (Fig. 5a). By screening for binding motifs within these ACRs, we found that the binding site of the GmLHY1a protein is enriched in the ACR located in the promoter region of *GmPIF4* (Fig. 5b).

To investigate how GmLHY1a regulates *GmPIF4* expression via these ACRs, we examined the potential regulatory effect of GmLHY1a on *GmPIF4* promoter activity using an in vitro electrophoretic mobility shift assay (EMSA) (Fig. 5c). Our results demonstrated a direct binding interaction between GmLHY1a and the motif within the *GmPIF4*-associated ACR. Furthermore, when the *Gmlhy1a/1b/2a/2b* mutants were analyzed, the chromatin accessibility at the *GmPIF4* loci was significantly altered, particularly at ZT36, where the *Gmlhy1a/1b/2a/2b* mutant exhibited a much more condensed chromatin to WT (Fig. 5d). We measured *GmPIF4* expression in *Gmlhy1a/1b/2a/2b* mutants and WT via RT-qPCR. We found that *GmPIF4* expression level in *Gmlhy1a/1b/2a/2b* mutant is lower than WT, which indicates that the GmLHY1a activates the *GmPIF4* expression in soybean (Fig. 5e). To validate the regulatory relationship, we expressed *GmLHY1a* using an estrogen inducible system in *Nicotiana*. RT-qPCR data revealed a significant increase in the expression level of the *GmPIF4p::LUC* following *β*-estradiol treatment (Fig. 5f).

**Figure 6:**
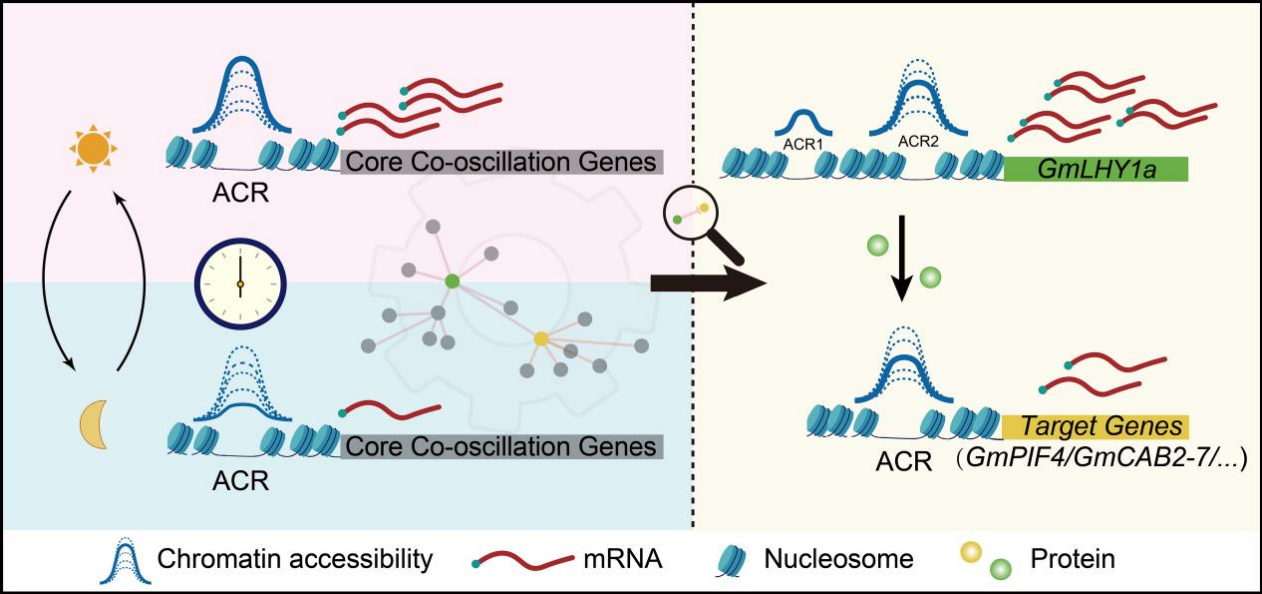
A general oscillating mechanism is proposed to highlight functional ACRs with rhythmic chromatin accessibility synchronize circadian inputs and outputs. On the left side, it shows that the CCOG genes exhibit resonance in chromatin accessibility and gene expression levels at different times of the day. Through a transcriptional regulatory network enriched based on ACRs, we identified transcriptional regulation on the right side. This includes the circadian rhythm of the core clock gene *GmLHY1a*, which is jointly regulated by 2 ACR segments in its promoter region, and these 2 ACRs maintain the stability of *GmLHY1a*'s circadian rhythm. Moreover, the GmLHY1a protein can regulate the transcription of downstream genes by binding to their ACRs, ultimately transmitting the circadian clock rhythm to downstream genes (i.e. *GmPIF4*, *GmCAB2-7*).

In summary, core clock genes such as *GmLUXs* or *GmLHYs* regulate diurnal changes in chromatin accessibility and bind to accessible regions of downstream genes, influencing their rhythmic expression. This study supports a model in which core circadian genes synchronize with exogenous environmental conditions via the TTFL, regulated by multiple epigenetic modifications (Nohales and Kay 2016). Both endogenous and exogenous diurnal rhythms are established, with core oscillator genes relaying rhythmic information downstream by binding to CRE marked by accessible chromatin and histone modifications. These processes regulate the transcription of target output genes in the same way (Fig. 6).

## Discussion

During dispersal to other latitudes, mutations at multiple core circadian clock genes have enabled soybean to adapt to light and temperature conditions across different latitudes, thereby facilitating yield improvement and expansion of its cultivation range (Lu et al. 2017, 2020; Dong et al. 2021). Previous studies have demonstrated that the expression of circadian clock genes and histone modifications exhibit synchronized circadian rhythms (Xue et al. 2023; Zhang et al. 2023), yet whether chromatin accessibility, as a fundamental epigenetic determinant of transcriptional regulation, exhibits similar rhythmicity in the circadian system remains unresolved.

In this study, we constructed a high-resolution multi-omics landscape by combing ATAC-seq, ChIP-seq and RNA-seq to investigate the oscillation of chromatin accessibility in soybean under free-running conditions. We found that soybean chromatin accessibility exhibits circadian rhythms, similar to histone modifications such as H3K9ac and H3K27ac, which oscillate in coordination with gene expression. By clustering CCOG, we found that the expression patterns of genes involved in processes such as light signaling, and stress resistance are also regulated by chromatin accessibility. Also, we found that downstream genes execute various functions, including regulating growth and developmental processes, gene expression, stomatal opening and closing, hormone responses, metabolism, and stress responses, ensuring temporal and spatial coherence with external environmental conditions.

Previous studies have shown that there are examples where core promoters and enhancers located far apart coordinately regulate *Flowering Locus T* gene expression in *Arabidopsis* (Liu et al. 2014). In this study, we found that *GmLHYs* genes possess relatively conserved CRE, with ACR1 and ACR2 serving distinct functions in soybean. *ACR1*, located at the distal region of the promoter, enhances *GmLHY1a* expression and functions as an enhancer, while *ACR2*, located proximally, is necessary for the rhythmic expression of *GmLHY1a*. We identified GmSPT and GmFBH3 as potential transcription factors that can bind to the *GmLHY1a ACR2* to regulate its transcription. Furthermore, the circadian clock genes *GmLHYs* and *GmLUX* can influence chromatin accessibility of the CCOG ACRs. When the core clock is lacking, the stability of circadian transcriptional feedback loops is disrupted, and chromatin modifiers (e.g. histone acetyltransferases, deacetylases, or chromatin remodelers) that control the temporal gating of chromatin accessibility in epigenetic regulations are disrupted, resulting in widespread, uncoordinated changes in genome-wide chromatin accessibility. Therefore, we propose that the transcription regulation through ACRs is a common pattern in circadian rhythm.

Furthermore, we found that the core circadian gene *GmLHY1a* regulates the *GmPIF4*, an output transcription factor of the circadian clock, by directly binding to its promoter through the ACR region. Elucidating circadian clock regulatory mechanisms enhances our understanding of the soybean circadian network. Moreover, it establishes a foundation for deciphering the molecular mechanisms underlying key biological processes regulated by the circadian clock, including photoperiod and temperate adaptability, light signal transduction, energy metabolism, and responses to biotic/abiotic stresses. In agriculture, soybean and other legumes form symbiotic nodules with rhizobia to fix nitrogen, thereby meeting their nutritional requirement for growth and development. It has been demonstrated that the circadian clock regulates the nodulation (Zhang et al. 2022b; Dong et al. 2023). Accordingly, we postulate that perturbations in circadian rhythmicity due to altered chromatin accessibility of core clock genes like GmLHY and GmLUX may represent an unrecognized regulatory node linking epigenetic dynamics to the temporal control of nodulation. As an integral part of this regulatory framework, this effort elucidates the epigenetic modification response components and regulatory networks governing the soybean circadian clock.

In recent years, gene editing targeting non-coding regions such as cis-regulatory elements have provided an approach to germplasm improvement (Hendelman et al. 2021; Lou et al. 2025). According to this time-course dependent cis-elements landscape in genome-wide targeted editing of cis-elements in the non-coding regions of soybean circadian clock genes holds significant scientific potential for resolving the trade-offs between phenotypic traits caused by gene pleiotropy. This approach enables the fine-tuning of phenotypes in a controlled and quantitative manner, which is particularly crucial for overcoming the long-standing negative correlation between flowering time and yield traits in traditional soybean breeding programs (Song et al. 2022; Aguirre et al. 2023).

In conclusion, our findings not only establish an epigenetics landscape of circadian regulation but also provide a perspective on how chromatin accessibility participates in transcriptional regulation and the function of chromatin accessible regions. This study investigated the CRE underlying circadian rhythms, thereby establishing a foundation for further exploration and utilization of CRE to improve agronomic traits.

## Materials and methods

### Plant materials

For collecting serial samples in free-running conditions, soybean (*Glycine max* (L.) Mer.) Williams 82 (Ws82) seedlings were grown under SD conditions (8 hL/16 hD) for about 18 d with temperature cycles of 24 °C (light, 300–500 μmol m^−2^ s^−1^)/22 °C (dark), until the first tri-foliate leaf opened. On the nineteenth day, the plants were transferred into LL (constant light, 24 °C). After 1 d under LL, the first tri-foliate leaf was sampled every 4 h for 20 h starting at ZT24 on the 20th day for multi-omics or qPCR under free-running conditions.

### Plasmid construction

The promoter region was amplified from Ws82 gDNA by PCR and in-fusion into a *0800-pGreen* vector to generate *GmPIF4p::LUC* (2,387 bp) and *GmLHY1ap::LUC* (2,571 bp). The full length of cDNA of transcription factor was amplified from Ws82 cDNA by PCR and in-fusion into a *62sk-pGreen* vector to generate *Gm35Sp::LHY1a*. This construct was introduced into *Agrobacterium tumefaciens* strain GV3101-pSOUP and used for *Nicotiana* transformation. For hairy root transformation, the *GmLHY1a* ACR was amplified by PCR from Ws82 gDNA and cloned into entry vector *Fu76*, *LUC* and *GUS* reporter were cloned into entry vector *Fu79* either. The destination vector *pSoy10* and recombinant entry vector were recombined using gateway LR (11791020, Invitrogen). The recombinant destination vectors were delivered into soybean hairy roots via *Agrobacterium rhizogenes* strain K599 mediated transformation. For protein purification, the *GmLHY1a* CDS without stop codon was cloned into *pET28a-His* vector by homologous recombination, then transformed into *E. coli* strain *BL21*.

### ChIP-seq library preparation

Soybean tissues were crosslinked with 1% formaldehyde and quenched with 0.2 M glycine. For each experiment, about 1 to 2 g of the sample was ground into a fine powder in liquid nitrogen. The nuclei were diluted in Honda buffer (0.44 M Sucrose, 1.25% Ficoll, 2.5% Dextran T40, 20 mM HEPES, 10 mM MgCl_2_, 0.5% Triton X-100, 5 mM DTT, and Protease inhibitor cocktail) at 4 °C. After nuclei lysis, chromatin was fragmented into 200 to 600 bp by ultrasound processing using a Bioruptor (Diagenode). The lysates were centrifuged at 16,000 *g* at 4 °C for 20 min. 40 μL suspended proteinG magnetic beads (1002D, Invitrogen) pre-washed with Antibody Buffer were added to the sonicated chromatin to pre-clean the chromatin. Chromatin immunoprecipitation was performed using 2 to 5 μL anti-H3K9ac (ab10812, Abcam) or anti-H3K27ac (07–360, Millipore). After incubating 8 h at 4 °C, the protein-DNA complexes were subjected to immunoprecipitation by incubating the antibody-bead complexes with the fragmented chromatin. Then, the target protein-DNA complexes were eluted from the beads by adding 1 mL of freshly prepared High salt buffer, Low salt buffer, LiAc buffer, and TE buffer. After reverse cross-linking of DNA and protein by Chelex-100, ChIP-DNA was extracted using phenol: chloroform: isoamyl alcohol. ChIP-DNA libraries were prepared using a NEBNext Ultra II DNA Library Preparation Kit (E7645, NEB). DNA clean beads (N411, Vazyme) were used to select 250- to 650-bp library fragments. Finally, the DNA fragments were sequenced using an Illumina HiSeq X-Ten system (paired-end 150-bp reads). 3 biological replicates were obtained for each time point.

### RNA-seq library preparation

Total RNA was isolated from mature leaves using the RNeasy Plant Mini Kit according to the manufacturer's recommendations (74904, QIAGEN). Ribosome RNA was removed from 0.5 to 1.5 g of Total RNA using a TruSeq Stranded Total RNA kit with Ribo-Zero Plant for RNA-seq (RS-122-2401, Illumina) according to the manufacturer's recommendations. RNA sequencing was performed on an Illumina HiSeq X Ten system (paired-end 150-bp reads). 3 biological replicates were obtained for each time point.

### ATAC-seq library preparation

Samples were collected every 4 h for 1 d (ZT24–ZT44) under constant light condition. Approximately 1 to 2 g of the soybean mature leaves were ground into a fine powder in liquid nitrogen. Then the crude nuclei were filtered with Miracloth. After nuclei extraction using Nuclei Protect Buffer (20 mM MOPS, 40 mM NaCl, 90 mM KCl, 2 mM EDTA, 0.5 mM EGTA, 0.5 mM Spermidine, 0.2 mM Spermine, 1 × PIC), 50 k isolated nuclei were prepared for chromatin tagmentation by Tn5 transposase (TD501, Vazyme) for 30 min at 37 °C. Digested chromatin was purified using a CTAB followed by PCR amplification, product purification (N411, Vazyme) and paired-end sequencing (2 × 150 bp) using an Illumina Hiseq X-Ten. 3 biological replicates were obtained for each time point.

### Data processing and analysis

For ATAC-seq, adapter sequences were removed using *Trimmonmatic* (version 0.39), and sequencing reads were aligned to the soybean reference genome (Gmax_Wm82_a2_v1) with *Bowtie2* (version 2.2.9) using the parameters: –very-sensitive –no-discordant –no-mixed –no-unal -k 10. Initial ACRs were identified using *MACS2* peak calling (macs2 callpeak -f BAMPE –keep-dup all -B –SPMR –nomodel –shift −100 –extsize 200, version 2.2.6) (Zhang et al. 2008; Bolger et al. 2014; Langmead et al. 2019). For each time point, we called peaks individually from 3 replicates (with downsampling to standardize depth) and from merged replicate reads, retained consistent peaks from both, then integrated ACRs across all time points to yield total ACRs.

Subsequently, the top 0.5% of ACRs with the highest signals were excluded, as were ACRs with RPKM (Reads Per Kilobase of transcript per Million mapped reads) <10 across all 6 time points. A threshold based on the 5th percentile (RPKM = 7) was applied to remove regions with consistently low accessibility. Using the variance in chromatin accessibility across the 6 time points, ACRs were further categorized into fluctuating ACRs (Coefficient of variation, CV ≥ 13, corresponding to the 10 percentile) and relatively stable ACRs (CV ≤ 13). The Clustering analysis was performed using *Fuzzy* C-Means Clustering.

The chromosomal distribution of ACRs was analyzed using *ChIPseeker* (version 1.26.2) with the TSS region parameter set to c (−1,000, 1,000) (Yu et al. 2015). For motif analysis, peak sequences were extracted and compared against random sequences using *MEME-ChIP*. Transcription factor enrichment was calculated using information from the PlantTFDB and JASPAR databases (Jin et al. 2017; Rauluseviciute et al. 2024). Enriched motifs were selected based on an adjusted *P*-value <0.00001 and %TP >10.

ChIP-seq analysis steps were conducted following the procedures described in ATAC-seq.

### RNA-seq data processing and analysis

Adapter sequences were trimmed using *Trimmonmatic* (version 0.39) and the processed reads were aligned to the soybean reference genome (Gmax_Wm82_a2_v1) using *HISAT2* (version 2.2.1). Gene expression levels were quantified with *StringTie*, and transcript abundances were normalized as TPM (Transcripts Per Million) (Kim et al. 2019). Genes with TPM values <1 across all time points were excluded.

Based on the variance of gene expressions across 6 time points, genes were classified into fluctuating genes (CV ≥ 16, corresponding to the 10 percentile) and relatively stable genes (CV ≤ 16). Clustering analysis was performed using *Fuzzy* C-Means Clustering, following the same procedure as for ATAC-seq.

For ACRs and gene expression datasets filtered and clustered in the previous steps, we utilized the *MFuzzy* package with the parameter min.acore = 0.3 (Kumar and Futschi 2007). This process resulted in 10,180 genes and 15,719 ACRs. Subsequently, a Spearman correlation coefficient threshold of ≥0.6 was applied, narrowing the results to 4,056 genes and 5,172 ACRs.

Gene Ontology (GO) enrichment analysis was performed using the *clusterProfiler* package (version 4.10.0) (Wu et al. 2021).

### Data visualization

All ATAC-seq, RNA-seq, and ChIP-seq bigWig files were generated using *deeptools* (version 3.5.5) bamCoverage with normalization to RPKM (Ramírez et al. 2016). Genome browser tracks were visualized using *pyGenomeTracks* (version 3.6) (Lopez-Delisle et al. 2021). Additionally, gene expression, ACR abundance, and ChIP peak-related plots were created in *R* (version 4.0.4) using the *ggplot2* package (version 3.3.5) (Ginestet 2011).

### Estrogen-inducible Assay

The *GmLHY1a* construct was generated by PCR amplification of Ws82 cDNAs, followed by recombination into the estrogen-inducible vector *pMDC7*. The *GmPIF4* promoter (−2,387 bp) was developed using the *pGreen-0800* backbone. The *Pro_EST_::GmLHY1a* (estrogen-inducible *GmLHY1a*) and *GmPIF4p::LUC* constructs were transformed into *Agrobacterium tumefaciens* strain GV3101, which were then co-infiltrated into *N. benthamiana* leaves. After 1 d of dark incubation, 30 μM β-estradiol or water was injected into the same infiltrated *N. benthamiana* leaves. At 12- and 24-h post-treatment, the leaves were harvested to extract RNA and quantify the expression of genes.

### Dual-luciferase reporter assay

The recombined reporter plasmid *pGreen-0800* and effector plasmid *pGreen-62sk* were introduced into *A. tumefaciens* strain GV3101 (pSoup). The reporter and effector plasmid were transiently co-transformed into *N. benthamiana* leaves. The firefly luciferase (LUC) and Renilla luciferase (REN) activities were quantified using Double-Luciferase Reporter Assay Kit (FR201, Transgen).

### Hairy root transformation

The constructed vector was transformed into *Agrobacterium rhizogenes* strain K599. The correct monoclonal colonies were shaken at 28 °C in LB medium until the OD_600_ reached approximately 0.5. The bacterial cells were collected by centrifugation at 5,000×g and resuspended in liquid Co-Cultivation medium (0.32 g/L B5, 30 g/L Sucrose, 0.59 g/L MES, 40 mg/L Acetosyringone, pH5.4). The Ws82 seeds were sterilized by Cl_2_ treatment for 14 h and imbibed overnight. Next day, remove the seed coat using a scalpel and cut off the radicle. After immersion and infection of explants with *Agrobacterium rhizogenes* for 1 h, the explants were retrieved, and excess bacterial solution was removed. The explants were then transferred to a Co-Cultivation medium (liquid Co-Cultivation medium, 4.25 g/L Agar) and incubated in the dark for 3 d. When the wound was enlarged, the explants were transferred to a hairy root induction medium (1/10× Gamborg B5, 30 g/L sucrose, 3.9 g/L MES, 4.25 g/L agar, 400 mg/L cysteine,154.2 mg/L DTT, 40 mg/L acetosyringone, pH5.4) and cultured under long-day conditions for 14 to 21 d. Those exhibiting dsRed fluorescent signals were identified as positive hairy roots.

### LUC signal capture

Transgenic hairy root was placed into plate with Co-Cultivation medium and 2.5 mM D-Luciferin (LUCK-100, GOLDBIO) with 0.01% Triton. After constant-dark treatment for 24 h, the LUC signal was captured for 96 h in constant dark condition by CCD camera. The *promoter::LUC* luminescence from 1 hairy root fragment every 2 h for 30 min.

### GUS staining

Transgenic hairy roots were vacuum infiltrated in pre-cooled 90% acetone for 5 min, rinsed with pre-cooled water and on ice for 20 min. They were subsequently incubated with GUS-staining solution (100 mM sodium phosphate, pH 7.0, 10 mM EDTA, 0.1% Triton X-100, 0.5 mM potassium ferrocyanide, 0.5 mM potassium ferricyanide, and 0.05% X-Gluc) at 37 °C overnight. The GUS-stained tissues were then cleared in 80% ethanol 3 times for more than 5 hours until the green pigmentation dissipated. The cleared tissues were observed directly under a microscope.

### Accession numbers

Sequence data from this article can be found in the Phytozome under the following accession numbers: *LHY1a* (Glyma.16G017400), *LHY1b* (Glyma.07G048500), *LHY2a* (Glyma.19G260900), *LHY2b* (Glyma.03G261800), *PIF4* (Glyma.08G303900), PRR5d (Glyma.07G049400), *LUX1* (Glyma.12G060200), *LUX2* (Glyma.11G136600).

## Supplementary Material

koag063_Supplementary_Data

## Data Availability

All data are available in the main text or the materials. The ATAC-seq (accession number:GSE276840), RNA-seq (accession number: GSE276845) and ChIP-seq (accession number:GSE276885) NGS data are available at NCBI GEO database.

## Dive Curated Terms

The following phenotypic, genotypic, and functional terms are of significance to the work described in this paper:

LHY Gramene: AT1G01060
LHY Araport: AT1G01060

CCA1 Gramene: AT2G46830
CCA1 Araport: AT2G46830

TOC1 Gramene: AT5G61380
TOC1 Araport: AT5G61380
