# Supplementary material for: Circadian oscillations in chromatin accessibility systematically orchestrate rhythmic gene transcription in soybean
Source: Plant Cell. 2026 Mar 9;38(4):koag063. doi: 10.1093/plcell/koag063 (PMC13102102; doi:10.1093/plcell/koag063)
Supplement: koag063_Supplementary_Data [file koag063_supplementary_data.zip › TPC-2025-0824R2__Supplementary Figures S1-S14.pdf]

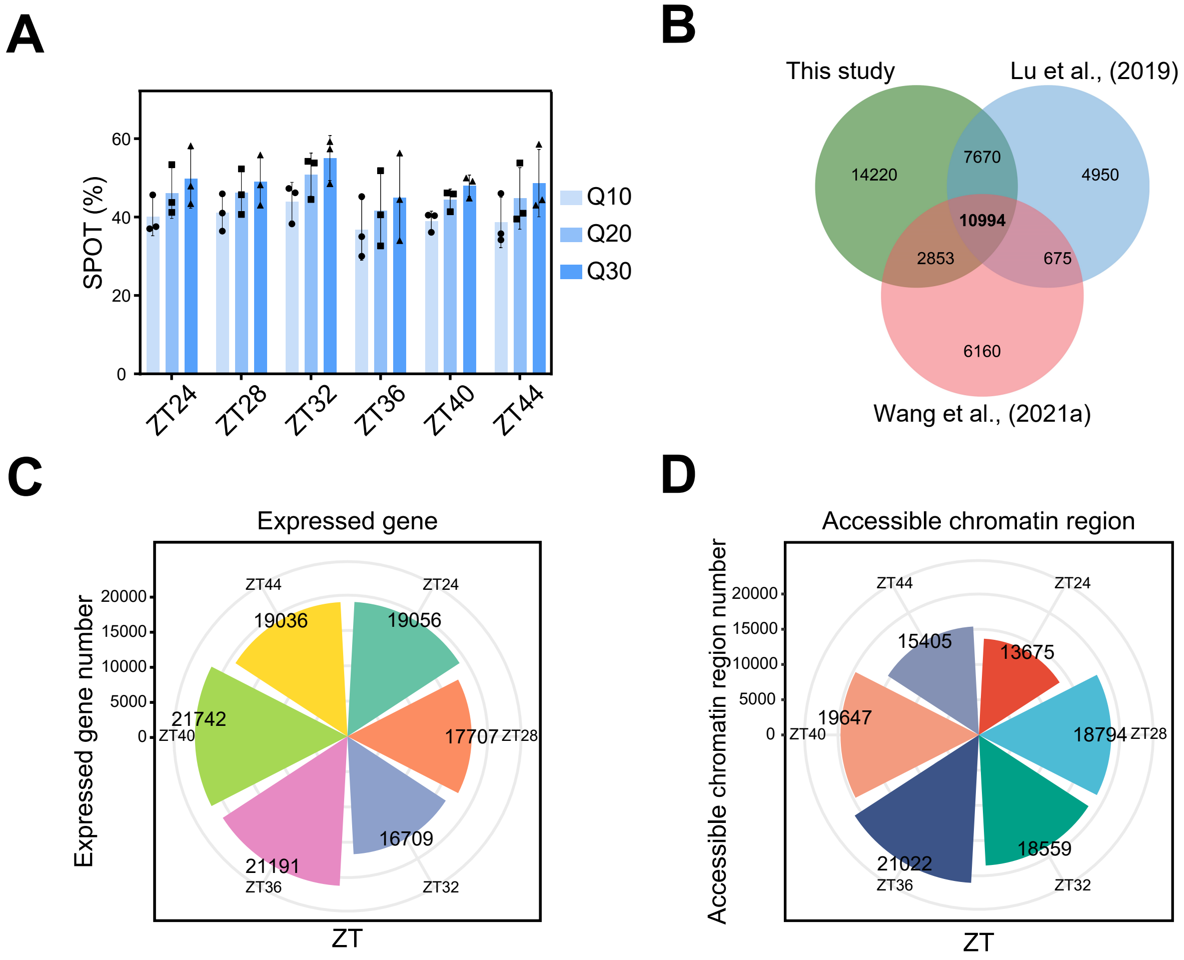

Supplemental Figure S1. Overview of ATAC-seq and RNA-seq data of soybean.
A: The SPOT ratio of ATAC-seq, Q10-Q30 represent the sequencing quality.
B: A Venn diagram showing the overlap of ACRs between time-course ATAC-seq and published single time point ATAC-seq.
C, D: Phase distribution of expression genes (B) and ACRs (C) under LL conditions. Each sector represents one ZT time point. The radius of each sector corresponds to the number of genes or ACRs expressed at that ZT point.

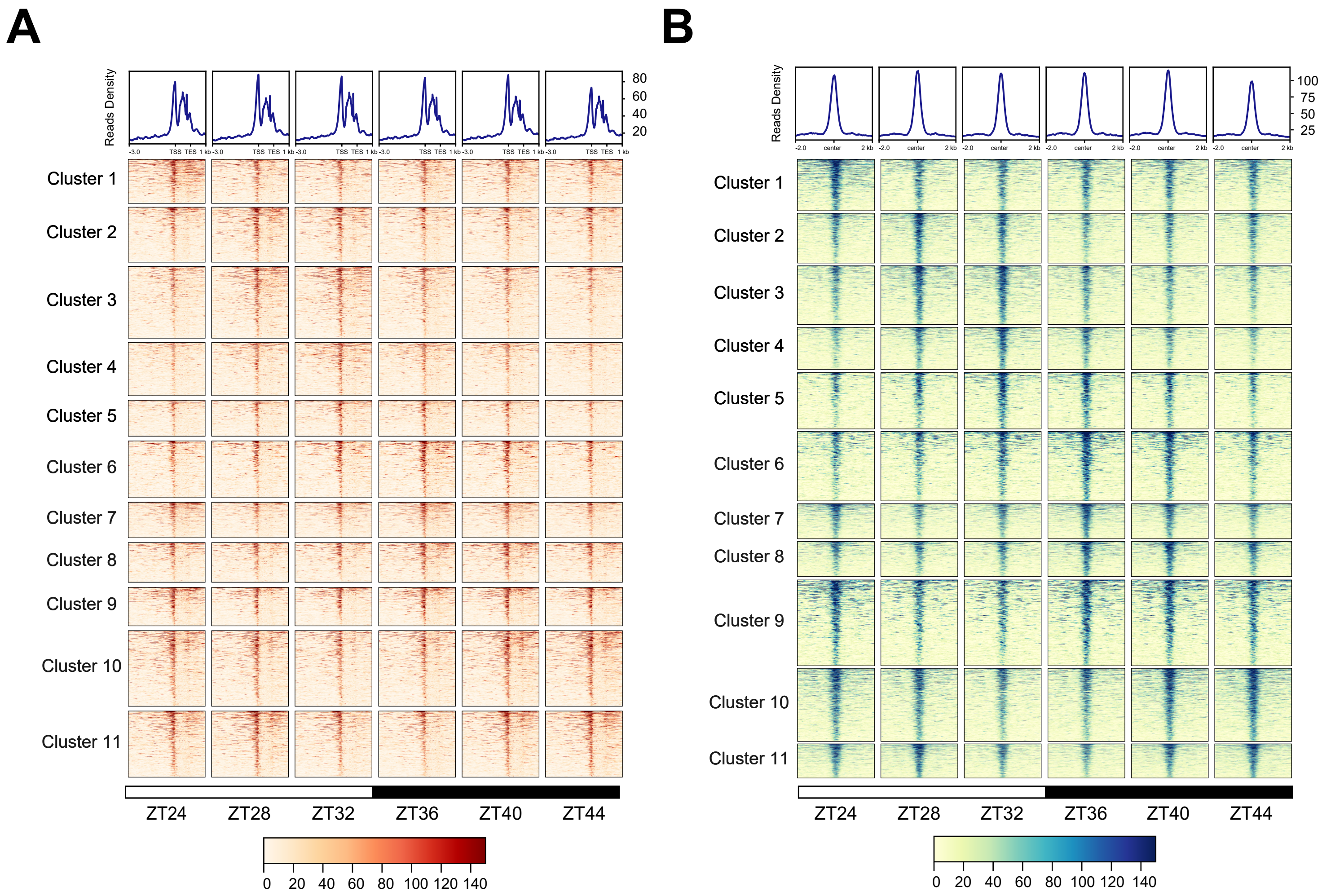

Supplemental Figure S2. CCOG oscillation patterns in different clusters.
A, B: Binding profiles of chromatin accessibility at the 3 kb upstream of TSS and 1 kb downstream of TES (A) and flank sequence of ACR summit (B). Heatmap of the chromatin accessibility of CCOG's ACRs sorted by the oscillation phase.

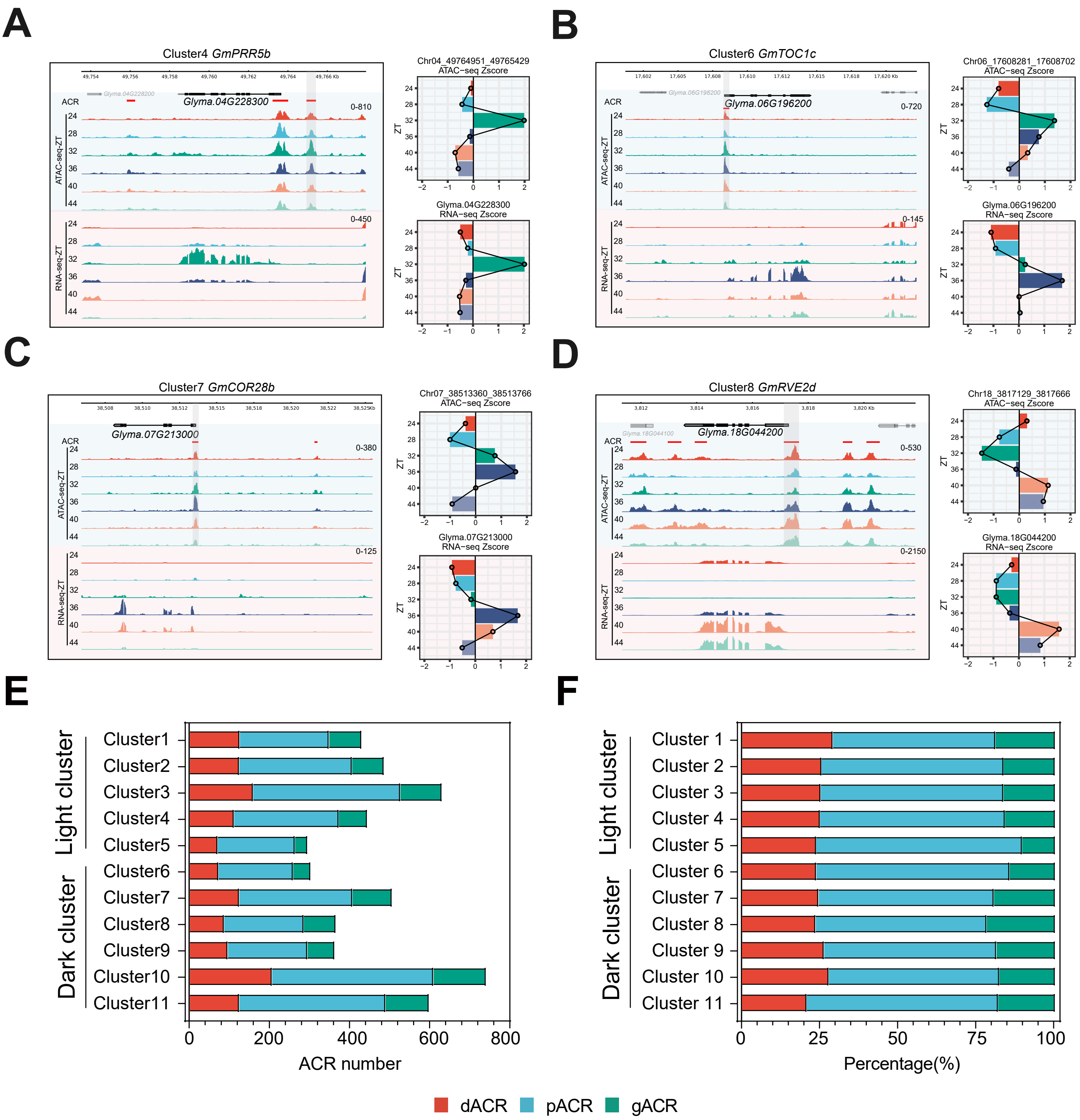

Supplemental Figure S3. Genome browser visualization of the chromatin accessibility oscillations in soybean clock genes.

A-D: ATAC-seq and RNA-seq genome tracks for *GmPRR5b* (A), *GmTOC1c* (B), *GmCOR28b* (C) and GmRVE2d (D) are shown on the left. The right panels display quantified chromatin accessibility of the shaded ACRs following Z-score normalization, with colors matching the six ZT time points in the genome tracks.

E, F: Distribution of ACR in CCOG categorized as distal ACR (dACR), proximal ACR (pACR), and genic ACR (gACR) are shown. dACR: ACRs located more than 1 kp away from the TSS or TES of a gene; pACRs fall within 1 kp of a gene's TSS and TES; gACR: ACRs located within the genebody, including 5' and 3'UTR. The number of ACRs in each category is indicated in the bar plots.

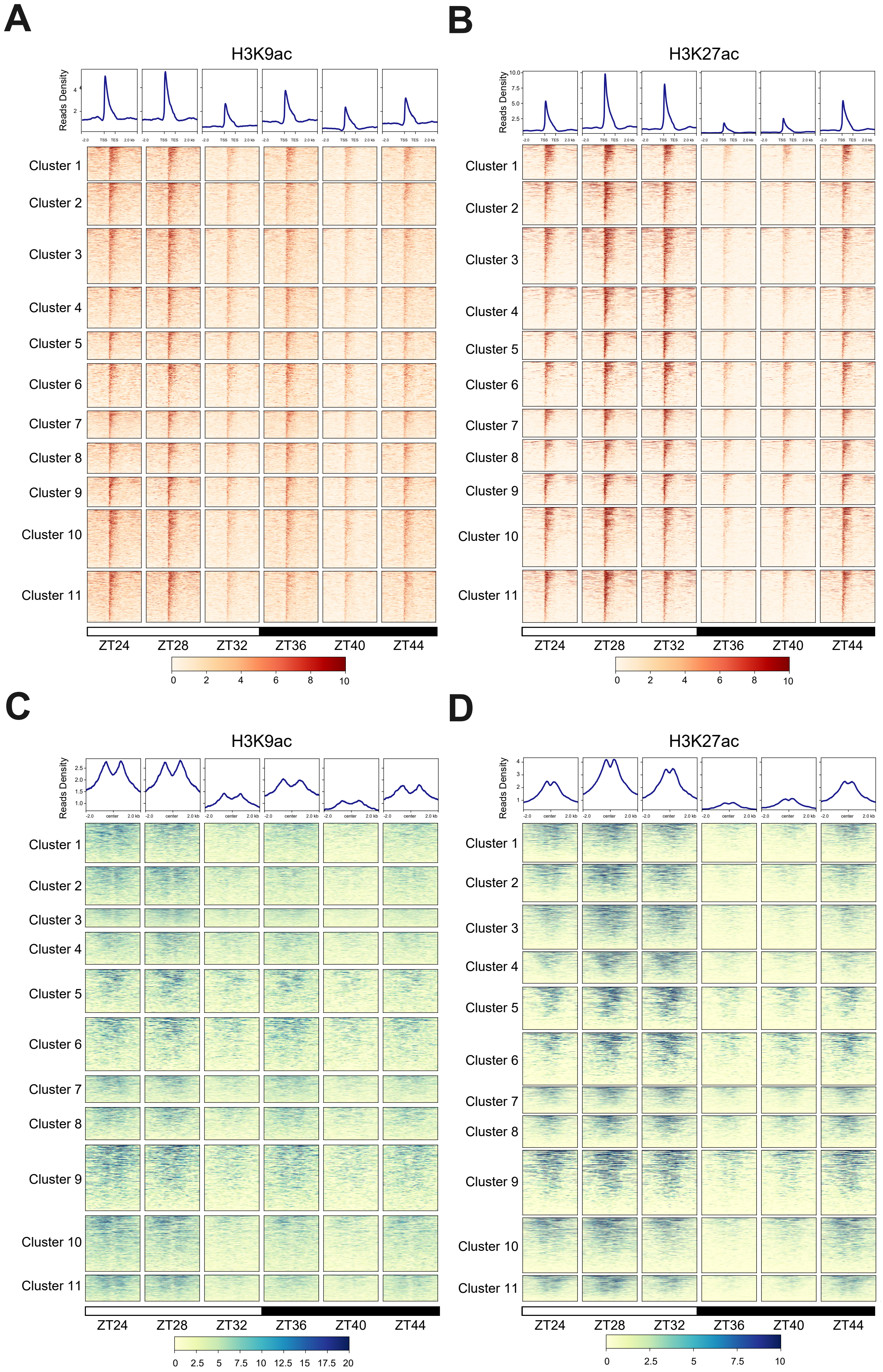

Supplemental Figure S4. Enrichment distribution of histone modification of CCOG. A-D: Binding profiles of H3K9ac (A, C) and H3K27ac (B, D) at the 2 kb upstream of TSS and 2 kb downstream of TES (A, B) and flank sequence of ACR summit (C, D). Heatmap of the chromatin accessibility of CCOG's ACRs sorted by the oscillation phase.

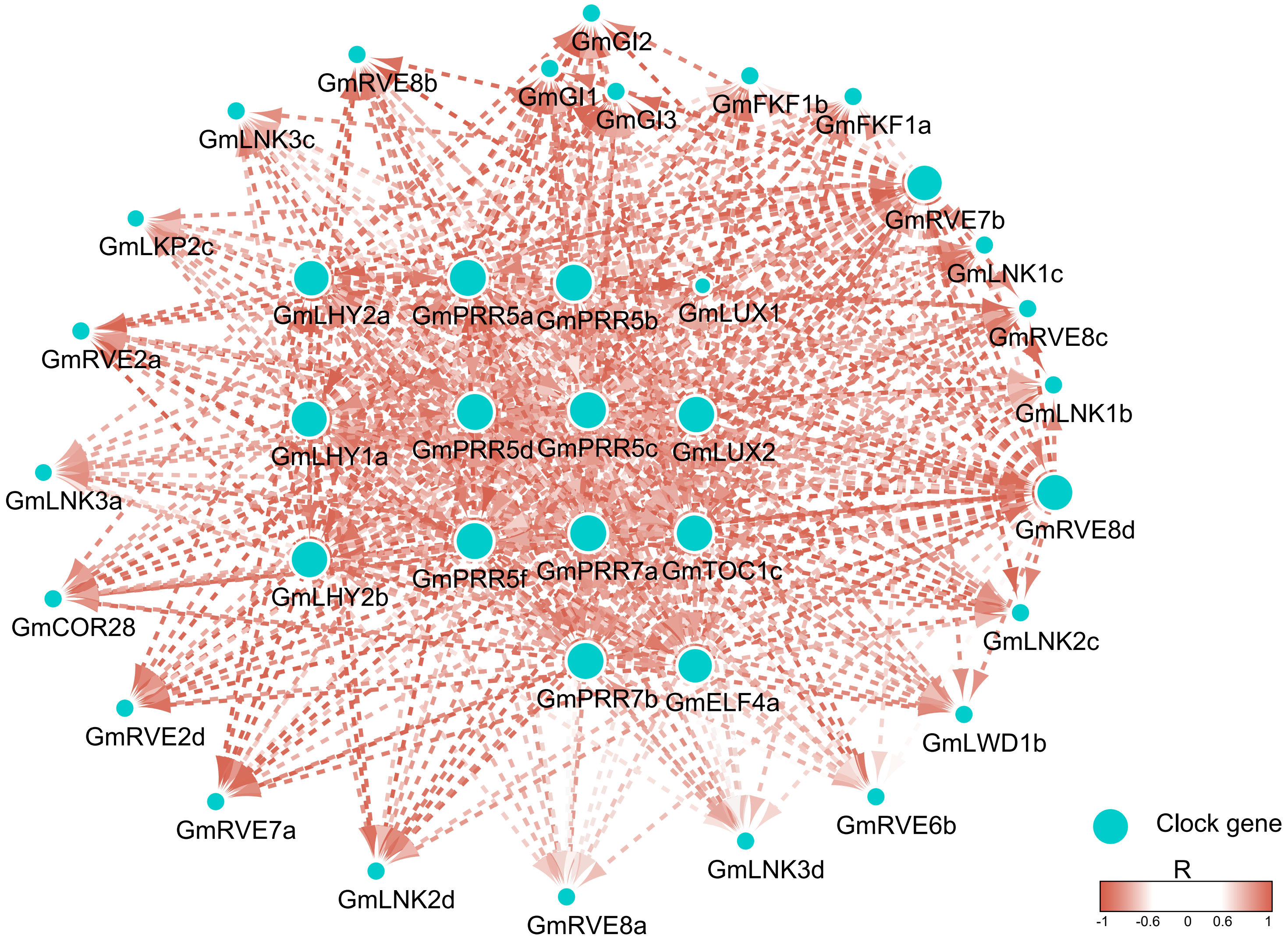

Supplemental Figure S5. Potential transcriptional regulatory network of core clock genes. Network of core clock genes. The size of point represents the frequency of potential binding, the dashed arrows represent the direction of regulation, and the intensity of the color indicates the correlation of gene expression between the two genes, which is R ≤-0.6 or R≥0.6.

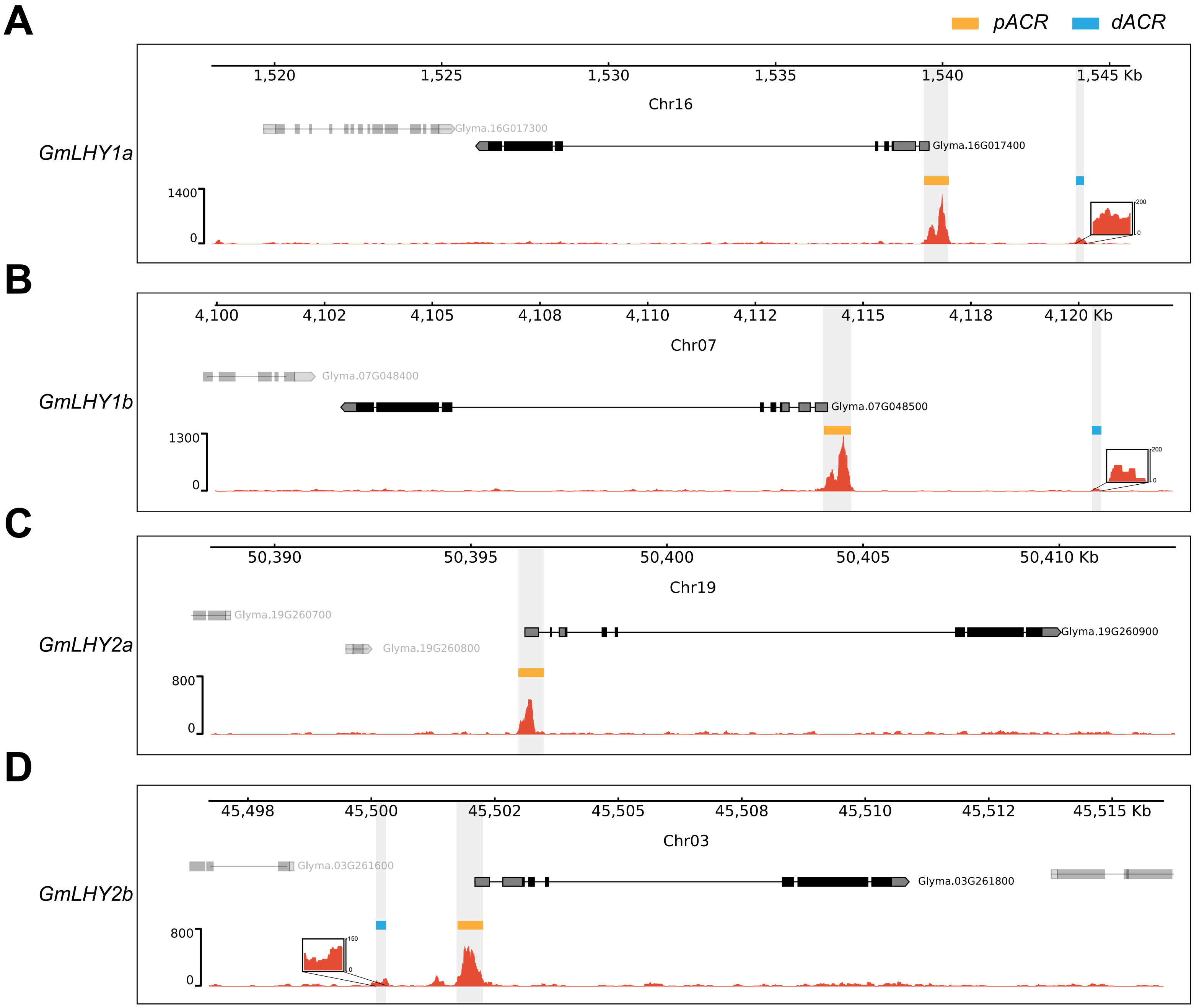

Supplemental Figure S6. The landscape of chromatin accessibility of *GmLHY*s locus in WT. A-D: The chromatin accessibility pattern of GmLHY homologous genes was shown. pACR (orange box) and dACR (blue box) are indicated. (A) *GmLHY1a*, (B) *GmLHY1b*, (C) *GmLHY2a*, and (D) *GmLHY2b*.

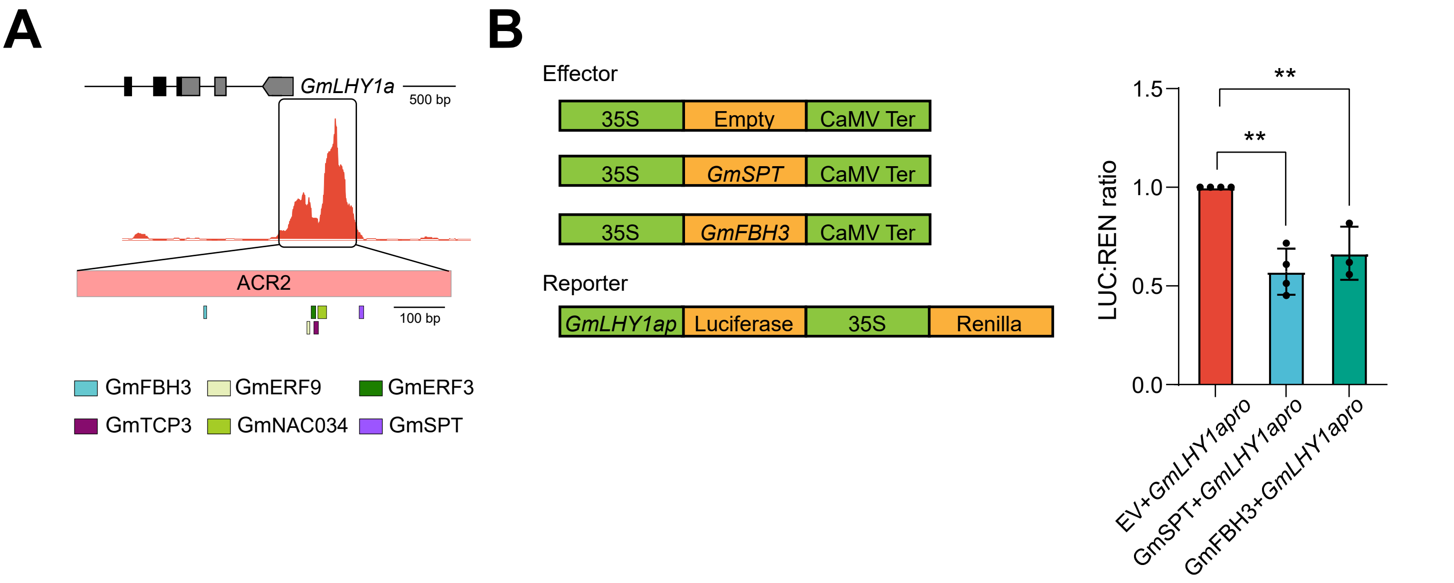

Supplemental Figure S7. The transcription factors binding the *GmLHY1a* promoter through the proximal ACR.

A: Location of the transcription factors protein-binding motif in the *GmLHY1a* promoter (2,571 bp, including *GmLHY1a ACR2*).

B: Constructs used for the transient transfection assay. CaMV Ter, Cauliflower Mosaic Virus terminator; LUC, luciferase; Renilla, Renilla Luciferase. Luciferase activity under control of *GmLHY1a* promoter showing the results from 3-4 independent replications; the value of each replication is represented by a dot. Data represent mean ± sd. A Student's *t*-test was used to generate the *p* values, ***p*<0.01.

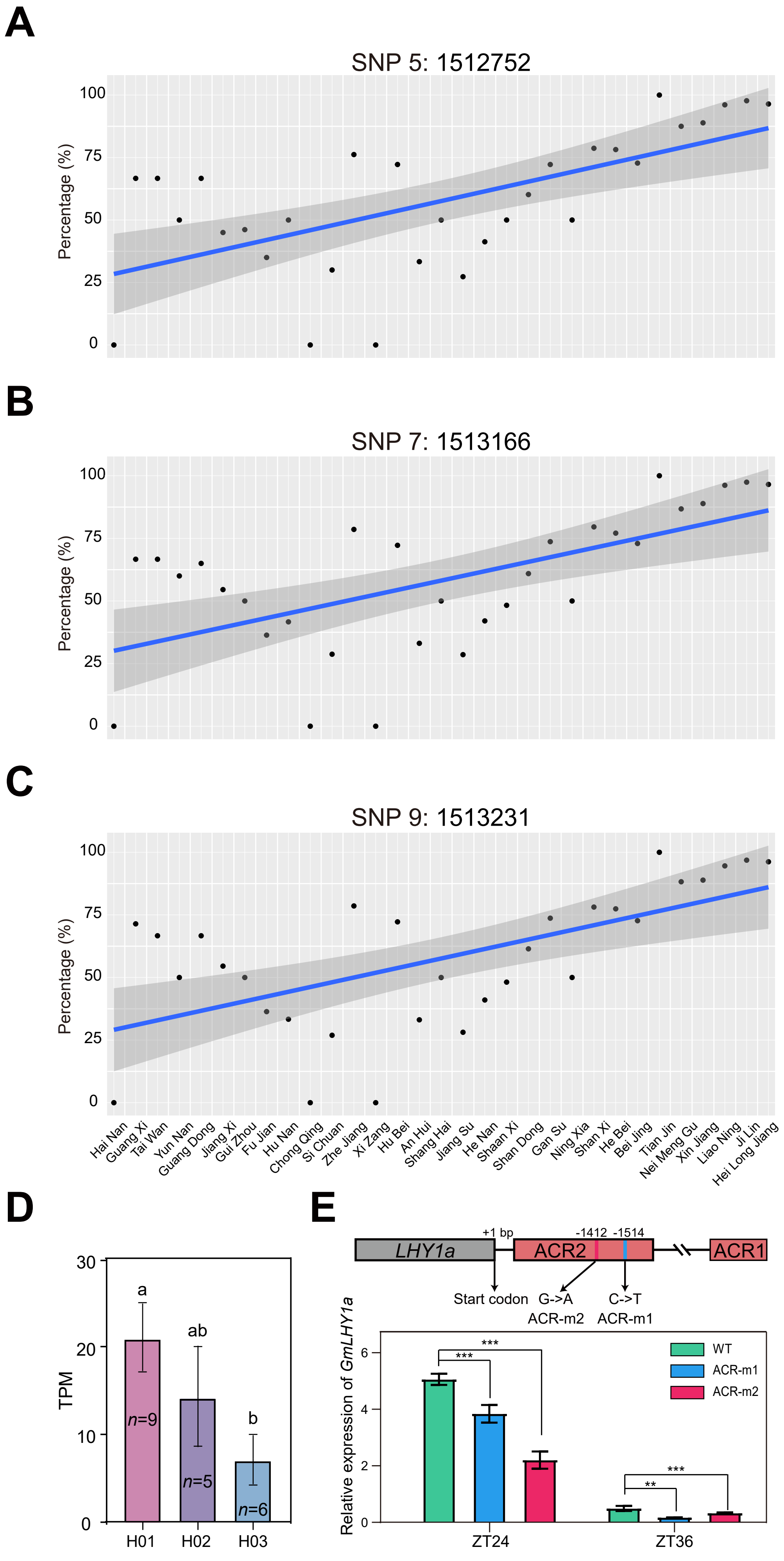
A
SNP 5: 1512752
Percentage (%)
0
25
50
75
100
B
SNP 7: 1513166
Percentage (%)
C
SNP 9: 1513231
Percentage (%)
Hai Nan
Guang Xi
Tai Wan
Yun Nan
Guang Dong
Jiang Xi
Gui Zhou
Fu Jian
Hu Nan
Chong Qing
Si Chuan
Zhe Jiang
Xi Zang
Hu Bei
An Hui
Shang Hai
Jiang Su
He Nan
Shaan Xi
Shan Dong
Gan Su
Ning Xia
Shan Xi
He Bei
Bei Jing
Tian Jin
Nei Meng Gu
Xin Jiang
Liao Ning
Ji Lin
Hei Long Jiang
D
TPM
0
10
20
30
a
ab
b
n=9
n=5
n=6
H01
H02
H03
E
LHY1a
+1 bp
ACR2
-1412
-1514
ACR1
Start codon
G->A
ACR-m2
C->T
ACR-m1
Relative expression of GmLHY1a
0
2
4
6
***
***
**
***
WT
ACR-m1
ACR-m2
ZT24
ZT36

Supplemental Figure S8. Footprints of selection for*GmLHY1a* ACR variants and the *GmLHY1a* expression level across accessions.

A-C: Proportion of the accessions (Total: 1979) with homologous haplotypes (A: SNP 5, B: SNP 7, C: SNP 9) in each distinct geography region, with ZH13 as reference genome. We sort Chinese cities by latitude, then calculate gene frequency of lines from each city.

D: *GmLHY1a* expression levels across 20 haplotypes (H01: Feng Di Huang, Ke Shan No.1, Zi Hua No.4, PI 578357, PI 398296, PI 549046, Zhu Twinning 2, PI 548362, Jin Dou No.23;H02: Hei He No.43, Shi Sheng Chang Ye, Dong Nong No.50, Ji Dou No.17, Tie Feng No.18; H03: Tong Shan Tian E Dan, 58 161, Han Dou No.5, Qi Huang No.34 , Xu Dou No.1, PI 562565). Expression values (TPM: Transcripts per million) are compared between haplotypes. The data are shown as mean ± SE. The number in each histogram represents the number of varieties having the relevant haplotype. Bars labeled with distinct lowercase letters (a, b, c) indicate statistically significant differences ($p<0.05$, Kruskal-Wallis with Dunn's multiple comparison test); shared letters denote non-significance.

E: The mutation sites of *GmLHY1a* ACR-m1 and *GmLHY1a* ACR-m2 are indicated. The expression levels of *GmLHY1a* were analyzed in ACR-m1 (NJAU0456) and ACR-m2 (NJAU1756)—EMS-induced point mutants carrying mutations within the ACR regions. Soybean seedlings were first grown under SD conditions at 24°C for 19 days, after which they were transferred to continuous light. Sampling was performed at ZT24 and ZT36, and *GmLHY1a* expression levels were measured via RT-qPCR, *GmACTIN* used as control, error bars represent standard error of three biological replicates. Statistical significance was determined using one-way ANOVA, **$p<0.01$, ***$p<0.001$.

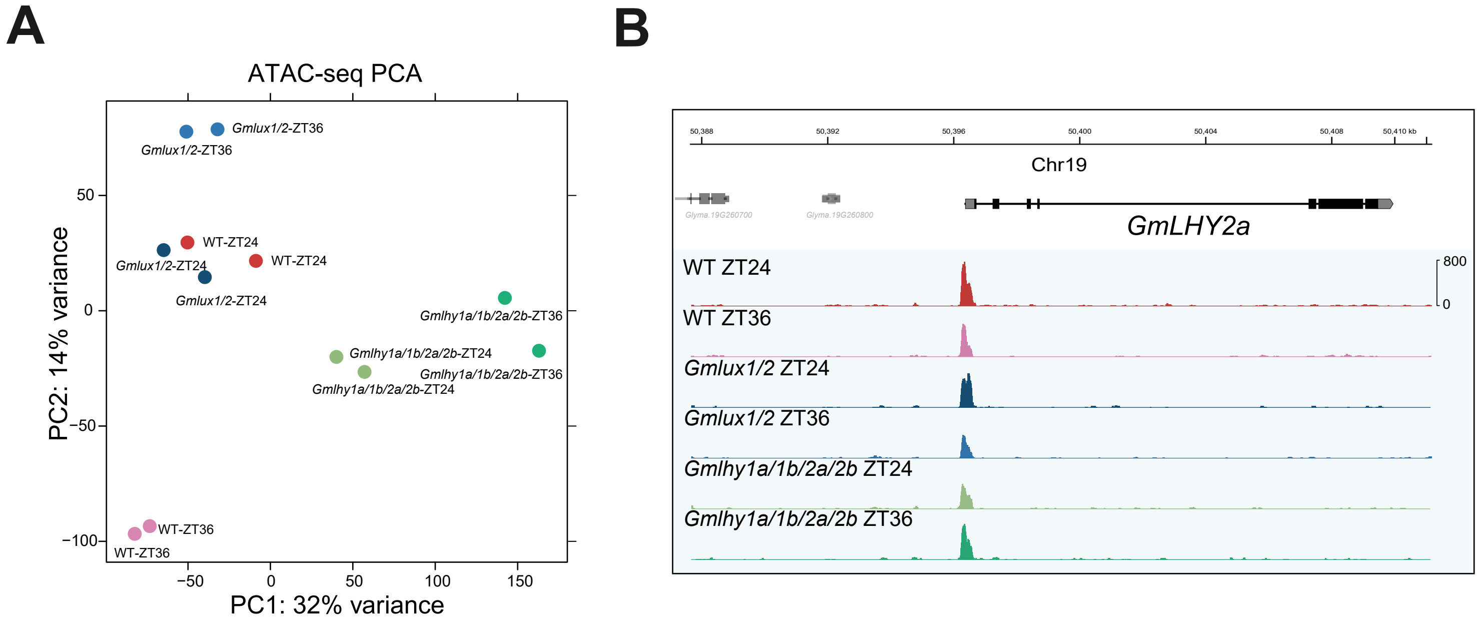

Supplemental Figure S9. Overview of chromatin accessibility in WT and mutants.
A: Principal component analysis (PCA) of chromatin accessibility was performed using ATAC-seq data from *Gmlux1/2*, *Gmlhy1a/1b/2a/2b*, and WT. The result demonstrated clear grouping and ordering of the 2 biological replicates per time point.
B: Representative ATAC-seq tracks in genomic regions of *GmLHY2a* showing the chromatin accessibility between time points and genotypes. The chromatin accessibility level was shown in the right.

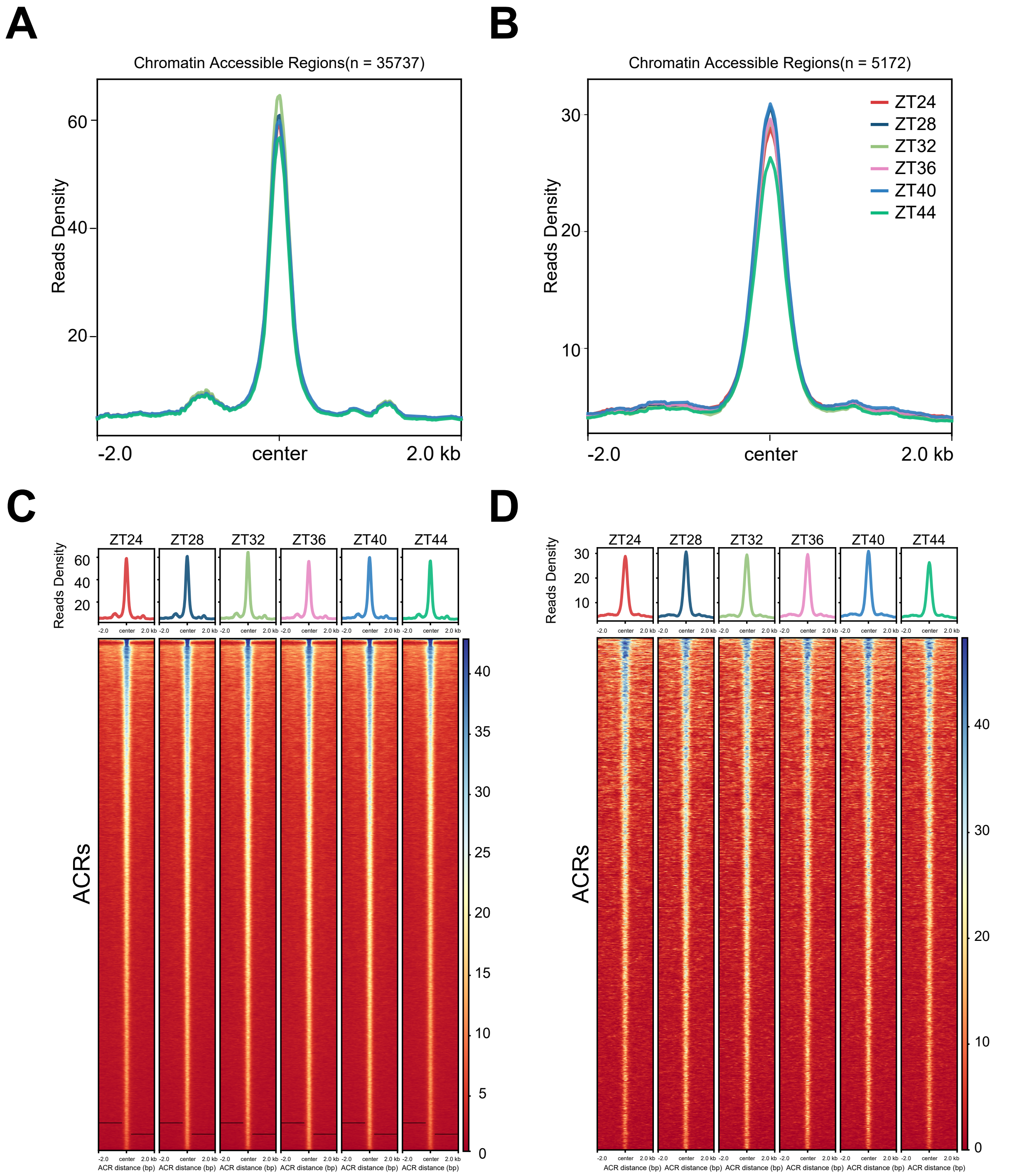

Supplemental Figure S10. The chromatin accessibility level of WT in CCOG.
A: Average chromatin accessibility of Ws82 ZT24-ZT44 on genome-wide ACR.
B: The average chromatin accessibility of Ws82 ZT24-ZT44 on CCOG ACRs.
C: Heatmaps showing the chromatin accessibility of each ACR and flanking 2 kb region of Ws82 ZT24-ZT44 on genome-wide ACRs.
D: Heat maps showing the chromatin accessibility level of Ws82 ZT24-ZT44 in each ACR and flanking 2 kb sequence on the CCOG ACRs.

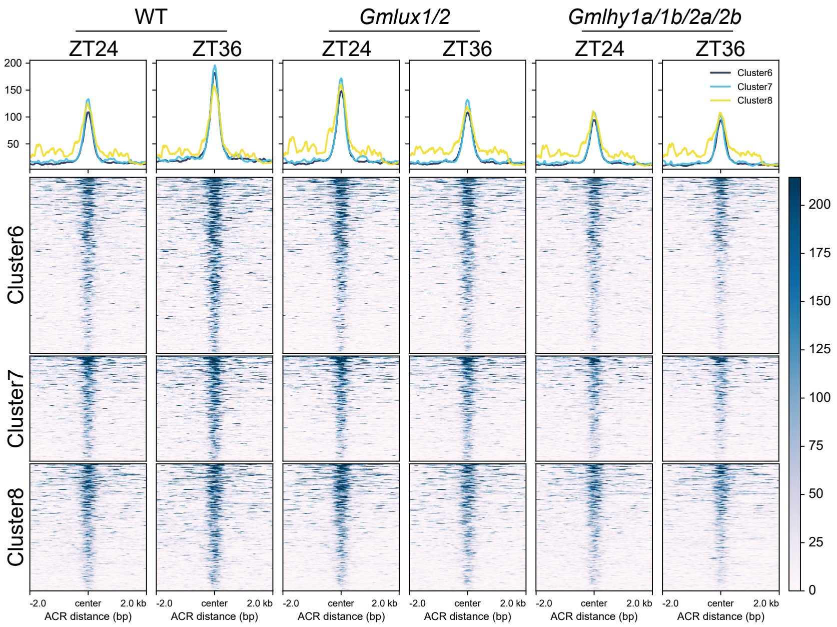

Supplemental Figure S11. The chromatin accessibility level of mutants in Cluster 6, 7 and 8.

Heatmaps showing the chromatin accessibility of ACR in CCOG and flanking 2 kb region of WT, *Gmlux1/2* and *Gmlhy1a/1b/2a/2b* at ZT24 and ZT36 in Cluster 6, 7 and 8.

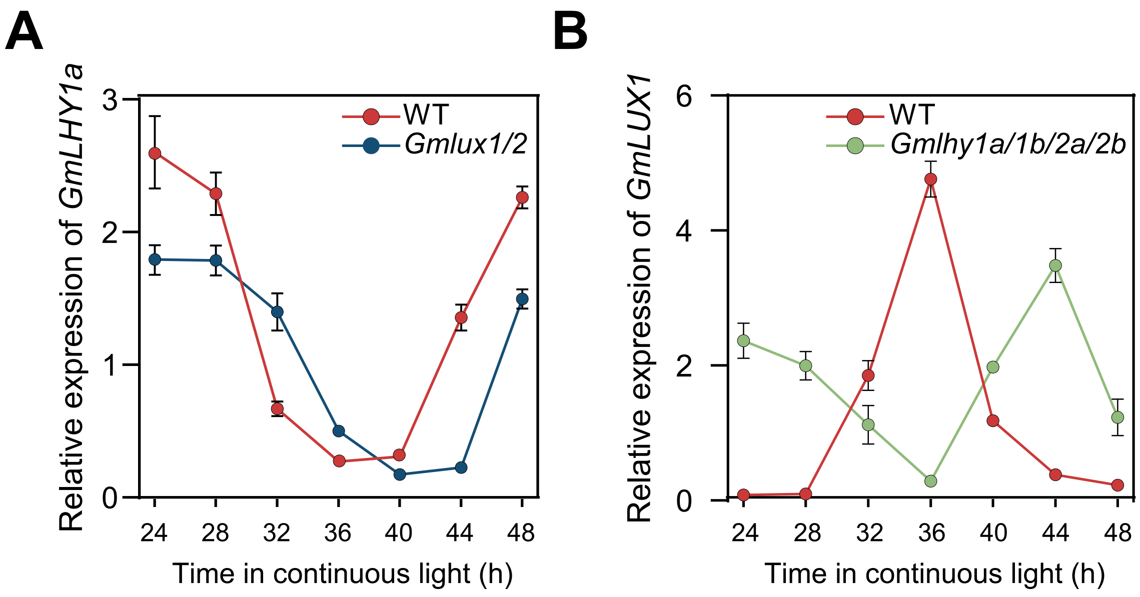

Supplemental Figure S12. The circadian expression patterns of *GmLHY1a* and *GmLUX1*.
A, B: The circadian rhythm of *GmLHY1a* and *GmLUX1* expression was measured in *Gmlux1/2* and *Gmlhy1a/1b/2a/2b* knockout mutants via RT-qPCR, *GmACTIN* used as control. Soybean seedlings were first grown under SD conditions at 24℃ for 19 days, after which they were transferred to continuous light. (A) *GmLHY1a* and (B) *GmLUX1*. Error bars represent standard deviation of three biological replicates.

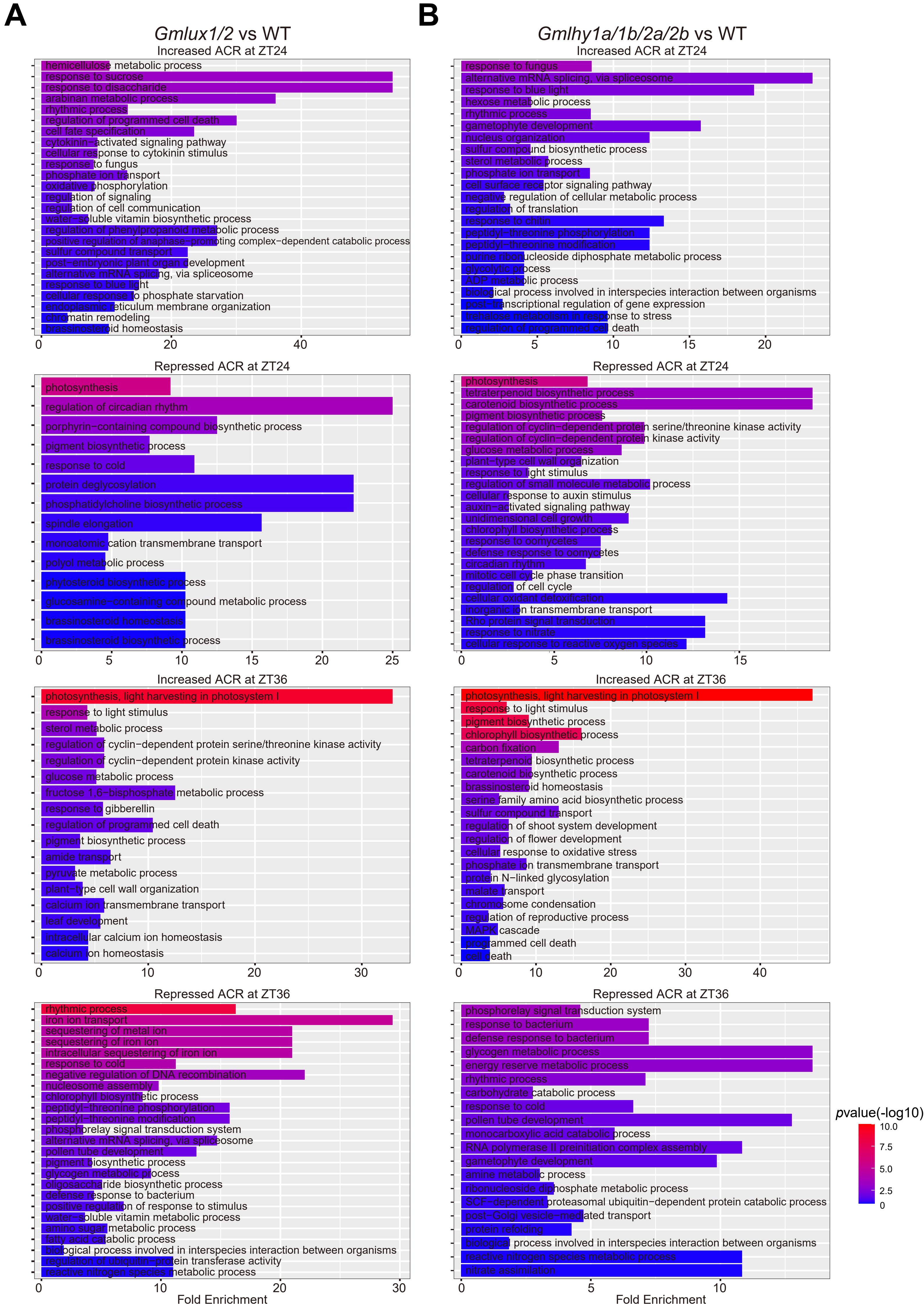

A
B
Gmlux1/2 vs WT
Gmlhy1a/1b/2a/2b vs WT
Increased ACR at ZT24
hemicellulose metabolic process
response to sucrose
response to disaccharide
arabinan metabolic process
rhythmic process
regulation of programmed cell death
cell fate specification
cytokinin−activated signaling pathway
cellular response to cytokinin stimulus
response to fungus
phosphate ion transport
oxidative phosphorylation
regulation of signaling
regulation of cell communication
water−soluble vitamin biosynthetic process
regulation of phenylpropanoid metabolic process
positive regulation of anaphase−promoting complex−dependent catabolic process
sulfur compound transport
post−embryonic plant organ development
alternative mRNA splicing, via spliceosome
response to blue light
cellular response to phosphate starvation
endoplasmic reticulum membrane organization
chromatin remodeling
brassinosteroid homeostasis
Increased ACR at ZT24
response to fungus
alternative mRNA splicing, via spliceosome
response to blue light
hexose metabolic process
rhythmic process
gametophyte development
nucleus organization
sulfur compound biosynthetic process
sterol metabolic process
phosphate ion transport
cell surface receptor signaling pathway
negative regulation of cellular metabolic process
regulation of translation
response to chitin
peptidyl−threonine phosphorylation
peptidyl−threonine modification
purine ribonucleoside diphosphate metabolic process
glycolytic process
ADP metabolic process
biological process involved in interspecies interaction between organisms
post−transcriptional regulation of gene expression
trehalose metabolism in response to stress
regulation of programmed cell death
Repressed ACR at ZT24
photosynthesis
regulation of circadian rhythm
porphyrin−containing compound biosynthetic process
pigment biosynthetic process
response to cold
protein deglycosylation
phosphatidylcholine biosynthetic process
spindle elongation
monoatomic cation transmembrane transport
polyol metabolic process
phytosteroid biosynthetic process
glucosamine−containing compound metabolic process
brassinosteroid homeostasis
brassinosteroid biosynthetic process
Repressed ACR at ZT24
photosynthesis
tetraterpenoid biosynthetic process
carotenoid biosynthetic process
pigment biosynthetic process
regulation of cyclin−dependent protein serine/threonine kinase activity
regulation of cyclin−dependent protein kinase activity
glucose metabolic process
plant−type cell wall organization
response to light stimulus
regulation of small molecule metabolic process
cellular response to auxin stimulus
auxin−activated signaling pathway
unidimensional cell growth
chlorophyll biosynthetic process
response to oomycetes
defense response to oomycetes
circadian rhythm
mitotic cell cycle phase transition
regulation of cell cycle
cellular oxidant detoxification
inorganic ion transmembrane transport
Rho protein signal transduction
response to nitrate
cellular response to reactive oxygen species
Increased ACR at ZT36
photosynthesis, light harvesting in photosystem I
response to light stimulus
sterol metabolic process
regulation of cyclin−dependent protein serine/threonine kinase activity
regulation of cyclin−dependent protein kinase activity
glucose metabolic process
fructose 1,6−bisphosphate metabolic process
response to gibberellin
regulation of programmed cell death
pigment biosynthetic process
amide transport
pyruvate metabolic process
plant−type cell wall organization
calcium ion transmembrane transport
leaf development
intracellular calcium ion homeostasis
calcium ion homeostasis
Increased ACR at ZT36
photosynthesis, light harvesting in photosystem I
response to light stimulus
pigment biosynthetic process
chlorophyll biosynthetic process
carbon fixation
tetraterpenoid biosynthetic process
carotenoid biosynthetic process
brassinosteroid homeostasis
serine family amino acid biosynthetic process
sulfur compound transport
regulation of shoot system development
regulation of flower development
cellular response to oxidative stress
phosphate ion transmembrane transport
protein N−linked glycosylation
malate transport
chromosome condensation
regulation of reproductive process
MAPK cascade
programmed cell death
cell death
Repressed ACR at ZT36
rhythmic process
iron ion transport
sequestering of metal ion
sequestering of iron ion
intracellular sequestering of iron ion
response to cold
negative regulation of DNA recombination
nucleosome assembly
chlorophyll biosynthetic process
peptidyl−threonine phosphorylation
peptidyl−threonine modification
phosphorelay signal transduction system
alternative mRNA splicing, via spliceosome
pollen tube development
pigment biosynthetic process
glycogen metabolic process
oligosaccharide biosynthetic process
defense response to bacterium
positive regulation of response to stimulus
water−soluble vitamin metabolic process
amino sugar metabolic process
fatty acid catabolic process
biological process involved in interspecies interaction between organisms
regulation of ubiquitin−protein transferase activity
reactive nitrogen species metabolic process
Repressed ACR at ZT36
phosphorelay signal transduction system
response to bacterium
defense response to bacterium
glycogen metabolic process
energy reserve metabolic process
rhythmic process
carbohydrate catabolic process
response to cold
pollen tube development
monocarboxylic acid catabolic process
RNA polymerase II preinitiation complex assembly
gametophyte development
amine metabolic process
ribonucleoside diphosphate metabolic process
SCF−dependent proteasomal ubiquitin−dependent protein catabolic process
post−Golgi vesicle−mediated transport
protein refolding
biological process involved in interspecies interaction between organisms
reactive nitrogen species metabolic process
nitrate assimilation
Fold Enrichment
Fold Enrichment
pvalue(-log10)
10.0
7.5
5.0
2.5
0

Supplemental Figure S13. The enriched pathway of differential ACR in clock gene's mutant. GO analysis showing enriched pathways of genes associated with increased and repressed ACRs in (A) *Gmlux1/2* and (B) *Gmlhy1a/1b/2a/2b* mutant. The *p* values were calculated by the Fisher's exact test.

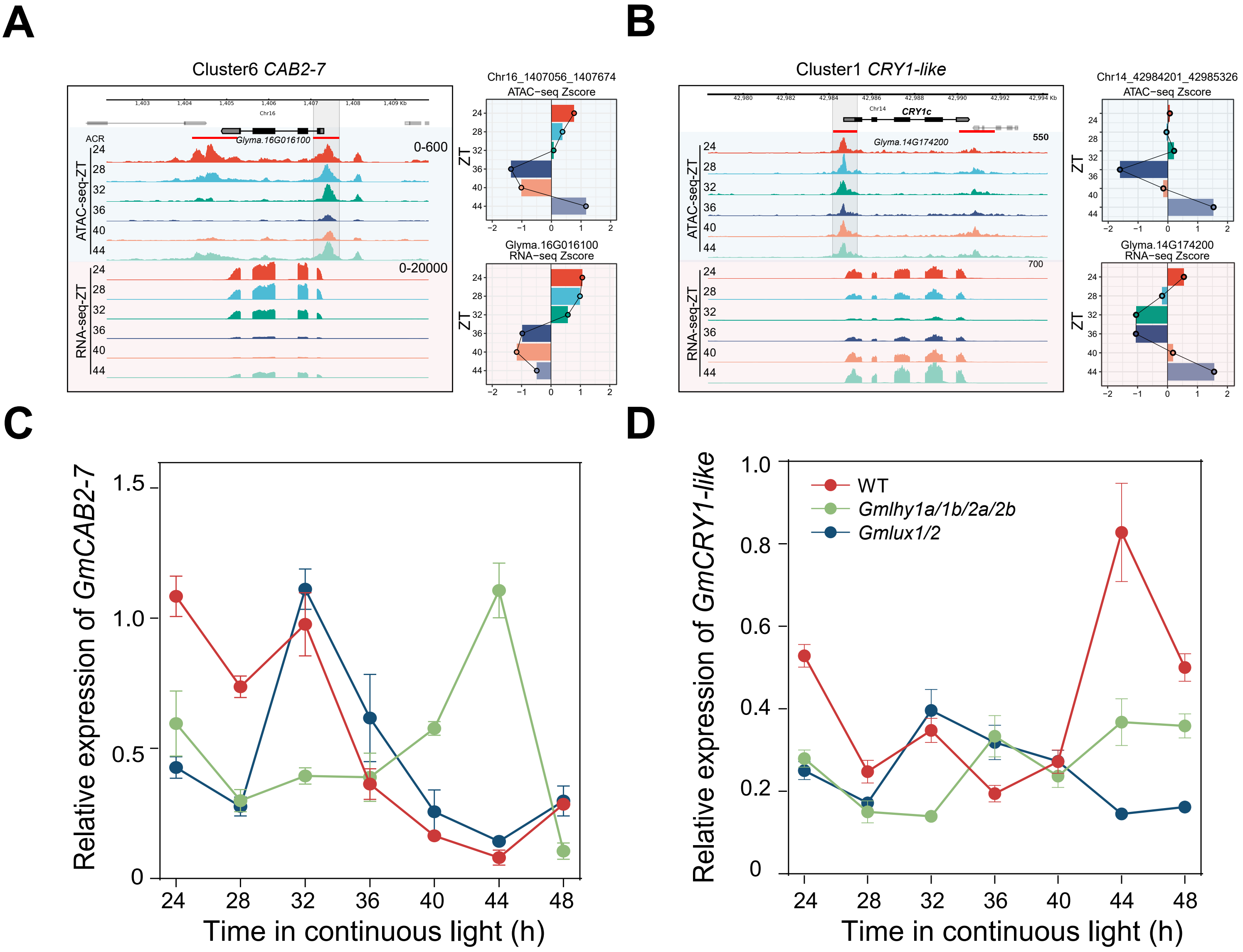
A
B
C
D
Cluster6 CAB2-7
Cluster1 CRY1-like
ACR
Glyma.16G016100
0-600
0-20000
ATAC-seq-ZT
RNA-seq-ZT
Chr16_1407056_1407674
ATAC−seq Zscore
Glyma.16G016100
RNA−seq Zscore
ZT
CRY1c
Glyma.14G174200
550
700
Chr14_42984201_42985326
ATAC−seq Zscore
Glyma.14G174200
RNA−seq Zscore
Relative expression of GmCAB2-7
Relative expression of GmCRY1-like
Time in continuous light (h)
WT
Gmlhy1a/1b/2a/2b
Gmlux1/2

Supplemental Figure S14. Chromatin accessibility and gene expression of *GmCAB2-7*and *GmCRY1-like*.

A, B: Genome browser track view of ATAC-seq profiles of chromatin accessibility and RNA-seq data at six time-points (ZT24, ZT28, ZT32, ZT36, ZT40, ZT44) in WT under free-running conditions. The red box represents the ACR. (A) *GmCRY1-like* and (B) *GmCAB2-7*.

C, D: The circadian rhythm of *GmCAB2-7* and *GmCRY1-like* expression was measured in *Gmlux1/2* and *Gmlhy1a/1b/2a/2b* knockout mutants via RT-qPCR, *GmACTIN* used as control. Soybean seedlings were first grown under SD conditions at 24°C for 19 days, after which they were transferred to continuous light. (C) *GmCAB2-7* and (D) *GmCRY1-like*. Error bars represent standard deviation of three biological replicates.
